# Major histocompatibility complex harbors widespread genotypic variability of non-additive risk of rheumatoid arthritis including epistasis

**DOI:** 10.1038/srep25014

**Published:** 2016-04-25

**Authors:** Wen-Hua Wei, John Bowes, Darren Plant, Sebastien Viatte, Annie Yarwood, Jonathan Massey, Jane Worthington, Stephen Eyre

**Affiliations:** 1 Arthritis Research UK Centre for Genetics and Genomics, Institute of Inflammation and Repair, Faculty of Medical and Human Sciences, Manchester Academic Health Science Centre, University of Manchester, Oxford Road, Manchester M13 9PT, UK; 2 Department of Women’s and Children’s Health, Dunedin School of Medicine, University of Otago, Dunedin 9016, New Zealand; 3 NIHR Manchester Musculoskeletal Biomedical Research Unit, Central Manchester NHS Foundation Trust, Manchester Academic Health Science Centre, Manchester, UK

## Abstract

Genotypic variability based genome-wide association studies (vGWASs) can identify potentially interacting loci without prior knowledge of the interacting factors. We report a two-stage approach to make vGWAS applicable to diseases: firstly using a mixed model approach to partition dichotomous phenotypes into additive risk and non-additive environmental residuals on the liability scale and secondly using the Levene’s (Brown-Forsythe) test to assess equality of the residual variances across genotype groups per marker. We found widespread significant (P < 2.5e-05) vGWAS signals within the major histocompatibility complex (MHC) across all three study cohorts of rheumatoid arthritis. We further identified 10 epistatic interactions between the vGWAS signals independent of the MHC additive effects, each with a weak effect but jointly explained 1.9% of phenotypic variance. *PTPN22* was also identified in the discovery cohort but replicated in only one independent cohort. Combining the three cohorts boosted power of vGWAS and additionally identified *TYK2* and *ANKRD55*. Both *PTPN22* and *TYK2* had evidence of interactions reported elsewhere. We conclude that vGWAS can help discover interacting loci for complex diseases but require large samples to find additional signals.

Genome-wide association studies (GWASs) have been extremely successful in highlighting loci associated with complex diseases such as rheumatoid arthritis (RA)^1^,^2^,^3^. While GWASs may continue to reveal novel loci based on the additive assumption, analyzing non-additive effects, such as gene-gene (GxG) and/or gene-environment (GxE) interactions, could provide novel insights into the underlying regulatory mechanisms^1^,^4^,^5^,^6^. Exploring GxG and/or GxE interactions in GWAS data has been attempted but, despite intensive efforts, the results so far have been relatively disappointing mainly due to low power of detection^7^,^8^,^9^. On the other hand, these results implicate that gene interactions could be prevalent but unlikely carry a big effect each and thus require a large sample to detect^6^,^9^. Currently available methods for detecting GxG interactions suffer from unmet computational and statistical challenges in supporting powerful meta-analysis of multiple cohorts^9^, and further potentially interacting environmental factors are not necessarily measured in study cohorts. Therefore, innovative approaches are needed to improve studying non-additive effects^10^.

One promising approach is genotypic variability based genome-wide association study (vGWAS) to search for loci with substantial genotypic variability as potential interaction signatures, i.e. differences of phenotypic variation across three SNP genotypes which tend to be small when only additive effects are important but large when non-additive effects such as interactions are important^11^,^12^. Such variability could be heritable to some extent and thus give rise to a new avenue of mapping genetic variants^13^,^14^. Hence, vGWAS has a great advantage in prioritizing potentially interacting loci without requiring prior knowledge of interaction types and interacting factors^15^,^16^. Additional explicit tests of GxE and/or GxG interactions are needed but only for the identified vGWAS loci, leading to a power increase attributed to a much reduced number of multiple tests^17^,^18^. Furthermore, studying biology of the identified vGWAS loci may inform what environmental factors should be further investigated. Therefore, vGWAS could be a cost effective approach to boost studies of GxG and GxE interactions based on existing GWAS data. However, vGWAS loci may not necessarily interact because other factors such as overdominance and scaling (i.e. various transformations) could also generate apparent genotypic variability^19^,^20^,^21^. Therefore, explicit tests of interactions and careful interpretation are essential to claim interacting signals.

There have been several successful applications of vGWAS in quantitative traits using either GWAS^18^,^19^,^22^ or gene expression data^17^,^23^,^24^. For example, Brown *et al*.^17^ reported abundant vGWAS signals within the major histocompatibility complex (MHC) region using RNA sequence data from lymphoblastoid cell lines derived from the TwinsUK cohort and subsequently confirmed GxE and GxG interactions underlying the signals. Yang *et al*.^19^ identified a well-known locus *FTO* in a vGWAS of body mass index in 170,000 samples from multiple GWAS cohorts, which is believed to interact with physical activity and/or lifestyle^25^. Intriguingly, the majority of the reported vGWAS loci are known major GWAS loci (i.e. with strong additive effects based on allelic means): *FTO* in body mass index^19^, *LEPR* in C-reactive protein levels and *ICAM1* in soluble ICAM1 levels^18^, *SLC2A9* in serum urate^22^, MHC in autoimmune diseases^5^. These observations led to a concern whether vGWAS might simply rediscover GWAS signals due to the mean-variance correlation, i.e. a distinct difference in allelic means likely accompanied by obvious genotypic variability in a locus^19^,^20^,^21^. Nevertheless, increasing evidence suggest that these major GWAS loci tend to have multiple roles and are indeed involved in interactions^26^,^27^,^28^,^29^, therefore it is biologically plausible to see them having signals in both GWAS and vGWAS analyses.

So far vGWAS has been little exploited in complex diseases probably because phenotypes classified either as a disease case or a healthy control provide little continuous variation. This problem can be remedied by applying a two-stage vGWAS approach: firstly using a mixed model method implemented in software such as GCTA^30^ to partition polygenic additive risk and non-additive environmental residuals for each sample on the liability scale^31^,^32^,^33^ and secondly using the Levene’s (Brown-Forsythe) test to assess equality of the residual variances across genotype groups per marker. It is therefore of interest to find out if vGWAS can also identify interacting loci in complex diseases. Here we report a pilot vGWAS of RA using a large GWAS cohort for discovery and two independent cohorts for replication. We aim to demonstrate that vGWAS is also useful to study disease traits.

## Materials and Methods

### Study cohorts and quality control (QC)

We used the Rheumatoid Arthritis Consortium International for Immunochip samples recruited in the UK (RACI-UK), US (RACI-US) and Sweden (RACI-SE, i.e. Swedish Epidemiological Investigation of Rheumatoid Arthritis) as described previously^2^. Each cohort was genotyped with the high density Immunochip^34^ in accordance with Illumina protocols and has been described in detail elsewhere^2^. Briefly, all RA patients fulfilled the 1987 criteria of the American College of Rheumatology and were tested for anti-citrullinated peptide antibody (ACPA) with a status of either seropositive (ACPA^+^), or seronegative (ACPA^-^) or unassigned. All participants provided written informed consent for participation. Further details of the sample collections in the three study cohorts can be found in the Supplementary Note by Eyre *et al*.^2^. This study was approved by the North West Research Ethics Committee (MREC 99/8/84). All experiments (e.g. genotyping) were performed in accordance with relevant guidelines and regulations.

We excluded SNPs on sex chromosomes and samples of non-European origin. RA cases that were either ACPA- or ACPA unassigned were also excluded in each cohort to avoid disease heterogeneity. A rigorous QC was conducted using PLINK^35^ for each cohort as described previously^2^ followed by additional QC based on the criteria suggested for accurate GCTA prediction of genetic risk^31^,^32^: minor allele frequency >0.01, SNP call rate >0.95, sample call rate >0.99, deviation from Hardy–Weinberg Equilibrium P < 1.0e-04.

### Two-stage vGWAS and Statistical analysis

**Figure 1:**
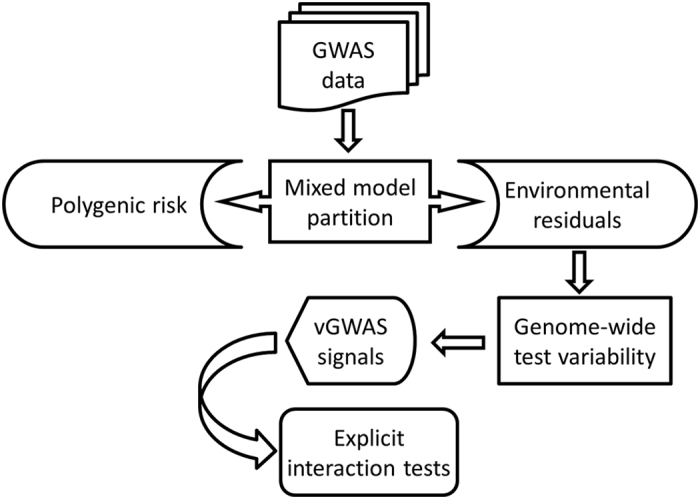
Workflow of the two-stage vGWAS approach with an extension of explicit interaction tests.

Figure 1 illustrates the workflow of the proposed two-stage vGWAS approach with an extension of explicit interaction tests. In this study GCTA was used to compute the genetic relationship matrix (GRM) and subsequently the first ten principal components (PCs) and then polygenic liability risk for each unrelated individual was predicted by imposing a GRM relatedness threshold of 0.15 recommended for Immunochip^33^ while setting the disease prevalence as 0.01 and fitting gender and the first 10 PCs as covariates in the mixed model. The resultant environmental residuals were tested for variance heterogeneity across three SNP genotypes using an R package VariABEL^36^ implemented the Levene’s (Brown-Forsythe) test that requires no assumption of normally distributed phenotypes:


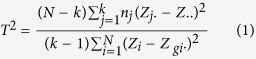


where the environmental residuals were the trait ***y***; ***Z***_***i***_** = |*****y***_***i***_ **-** ***ỹ***_***gi***_**|** is the deviation of ***y*** of the ***i***^***th***^ sample (***y***_***i***_) and the median of ***y*** in samples with genotype ***g*** (***ỹ***_***gi***_); ***N*** is the sample size and ***k*** is the total possible genotypes; ***n***_***j***_ is the number of samples with genotype ***j***; ***Z***_***j***_. is the mean deviation from the median for genotype ***j*** and ***Z***. is the mean deviation from the overall median. When ***N*** is large, ***T***^***2***^ is an approximate χ^2^ test taking two degrees of freedom.

To be extra cautious about the impact of normality, we also compared the vGWAS results based on the raw environmental residuals with those based on fully normalized environmental residuals using rank transformation available in the GenABEL package^37^. We found the normalization led to overly conservative vGWAS results in RACI-US and RACI-SE (Supplementary Figure S1) and thus reported only the results based on the raw environmental residuals.

We derived the significance threshold as 2.5e-05 based on permutation with 1000 iterations where a vGWAS was carried out using the randomly permutated environmental residuals and the lowest P value was recorded in each iteration (Supplementary Figure S2). We performed vGWAS in each of the three cohorts and used RACI-UK as the discovery cohort and examined any vGWAS significant SNPs for direct replication in RACI-US and RACI-SE, i.e. the same SNP with a vGWAS P value less than 5.0e-02.

For comparison we also performed a conventional GWAS for each cohort using PLINK and following the previous study^2^ that used a subset of independent common SNPs (i.e. minor allele frequency >0.05 and minimum inter-SNP linkage disequilibrium (LD)) to compute GRM and subsequently the first 10 PCs. A forward selection approach was applied to the genome-wide significant (P < 5.0e-08) GWAS SNPs identified in RACI-UK iteratively to identify a set of independent signals to represent the additive background. In each iteration the logistic regression model fitting the covariates and any pre-selected SNPs was used to test all remaining SNPs and select the most associated SNP for next round until none had a P value less than 5.0e-02.

We tested interactions between the identified vGWAS SNPs in RACI-UK as follows: a) PLINK was used to compute pairwise LD (in r^2^) for all the identified SNPs and then only pairs of SNPs in low LD (i.e. r^2^ < 0.1)^9^ were selected for interaction tests; b) the logistic regression model was used to test interactions for each of the ***k*** selected pairs of SNPs by fitting a pair of SNPs and their interaction terms as well as covariates of gender and the first 10 PCs derived from the GRM based on the subset of independent SNPs, with a significance of threshold defined as 0.05/(k^*^(***k***-1)/2); c) significant pairwise interactions were assessed while conditioning on the additive background of independent GWAS signals and unique pairs of interactions were identified using the forward selection approach above. Variance explained by the selected epistatic SNP pairs was calculated using a full logistic regression model fitting all the covariates, the additive background and the independent pairs of SNPs and their interactions. The identified epistatic SNP pairs were tested for replication individually in RACI-US and RACI-SE and the total variance explained was calculated following the approach above.

We further merged the three study cohorts into one and then performing the two-stage vGWAS as well as GWAS adjusted for cohort and the covariates described above to explore the impact of increased sample size. After the QC and GCTA analysis the combined cohort had 18,405 unrelated samples (5954 cases and 12,451 controls) and 107,144 SNPs in the vGWAS and GWAS. We quoted SNP genomic locations in the GRCh38/hg38 version throughout.

## Results

**Figure 2:**
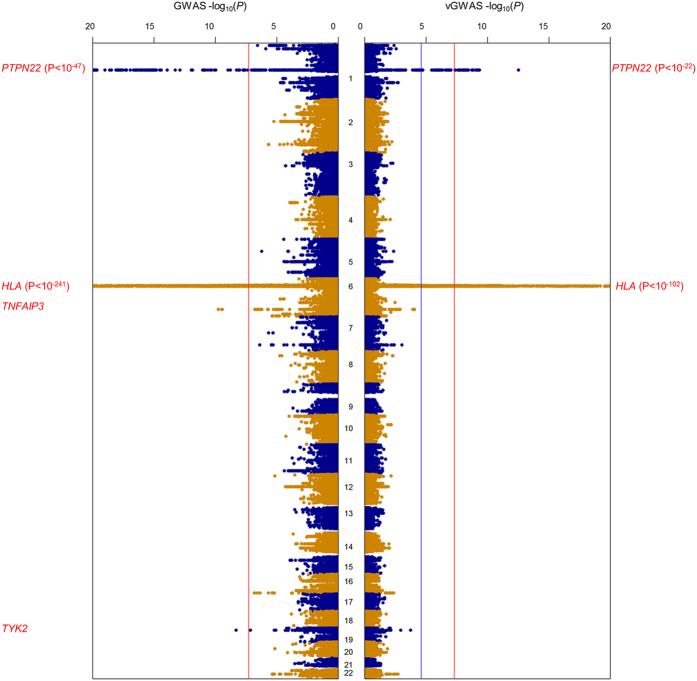
Aligned Manhattan plots of GWAS (left) and vGWAS (right) analyses in RACI-UK. P values are at the -log_10_ scale; red line represents GWAS genome-wide significance threshold; blue line represents vGWAS significance threshold derived from permutation; significant loci are annotated to genes in red (or blue if reached only the vGWAS significance threshold).

The vGWAS analysis identified 1639 significant SNPs (P < 2.5e-05) in the RACI-UK cohort where the Immunochip-wide heritability of ACPA^+^ RA was estimated as 24.7% (Table 1). About 92% of the identified vGWAS SNPs mapped to MHC and the remaining mapped to *PTPN22* – both were major RA associated loci in the conventional GWAS that in addition identified *TNFAIP3* and *TYK2* at the genome-wide significance level (P < 5.0e-08) (Fig. 2). Forward selection of the significant GWAS SNPs led to an additive background composed of 20 independent SNPs (Supplementary Table S1). The quantile-quantile (QQ) plots of both the vGWAS (Supplementary Figure S1) and GWAS (Supplementary Figure S3) analyses in RACI-UK showed no sign of inflation.

In contrast, the vGWAS analyses detected only 154 SNPs in RACI-US (heritability estimated as 24.6%) and 343 in RACI-SE (heritability estimated as 18.5%) under the same significance threshold, suggesting the two independent cohorts were underpowered probably due to the relatively small samples used (Table 1). The QQ plots for RACI-US and RACI-SE indicated slight deflation in the vGWAS analyses (Supplementary Figure S1) but not in the GWAS analyses (Supplementary Figure S3). Nearly all the vGWAS SNPs identified in both RACI-US and RACI-SE also mapped to MHC, except for rs2322659 (P = 7.7e-06) mapped to *LCT* in RACI-US (Supplementary Figures S4 and S5).

We found that a quarter (i.e. 413 out of 1639) of the identified vGWAS SNPs in RACI-UK had direct replication in both the RACI-US and RACI-SE cohorts (Supplementary Table S2) and they were all within the MHC region spanning the Class III and Class II. In addition, the two top vGWAS SNPs rs2476601 (P = 6.7e-22) and rs6679677 (P = 1.4e-21) within *PTPN22* in RACI-UK each had a direct replication only in RACI-SE with a P value of 3.3e-02 and 3.0e-02 respectively, suggesting genotypic variability within the locus was detectable only with a large sample size. The top vGWAS SNP rs2322659 within *LCT* in RACI-US had no replication in either RACI-UK or RACI-SE.

**Figure 3:**
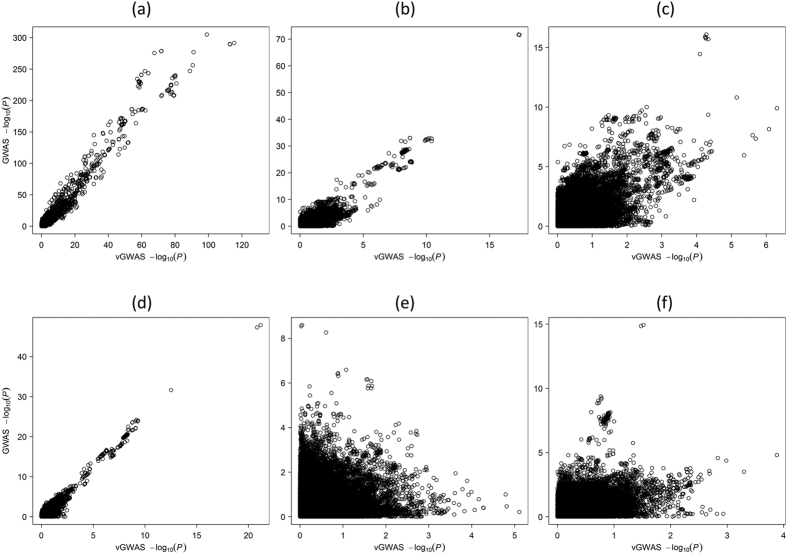
Concordance plots of GWAS P values against vGWAS P values. (**a**) the Combined cohort using all SNPs; (**b**) the Combined cohort excluding SNPs within the MHC region; (**c**) the Combined cohort excluding SNPs within the MHC and *PTPN22* regions; (**d**) the RACI-UK cohort excluding SNPs within the MHC region; (**e**) the RACI-US cohort excluding SNPs within the MHC region; (**f**) the RACI-SE cohort excluding SNPs within the MHC region.

Interestingly, in RACI-UK the most strongly associated vGWAS SNP rs1964995 (intergenic between *HLA-DRA* and *HLA-DRB5*) was about 134 kb away from the most strongly associated GWAS SNP rs6931277 (near *HLA-DRB1*) (Fig. 3a), and the two SNPs were also the top vGWAS and GWAS signals respectively in both RACI-US and RACI-SE (Supplementary Table S2). These results (re)confirm that the MHC region harbors both strong additive and non-additive effects^29^,^38^ and suggest that the top non-additive sites could differ from the top additive loci. Nevertheless, the top GWAS SNP rs6931277 could also be involved in interactions as it had a strong vGWAS signal (i.e. P = 1.2e-96, 2.9e-07 and 7.1e-11 in RACI-UK, RACI-US and RACI-SE respectively).

We tested interactions for 11,871 pairs of the RACI-UK significant vGWAS SNPs with r^2^ < 0.1. Based on a significance threshold of 4.2e-06 derived from Bonferroni correction, we found a large number of significant interactions all within MHC, of which 2,962 pairs of SNPs had a conditional interaction P value between 3.4e-07 and 5.0e-02 independent of the additive background. Applying forward selection to the 2,962 pairs and requiring a conditional interaction P < 1.0e-03 in each step, we identified 10 independent epistatic pairs of SNPs that spanned the entire MHC region and were replicated in both RACI-US and RACI-SE except for the rs9366778 - rs2239707 pair in RACI-US (Table 2, Supplementary Figure S6). In contrast to the additive background that explained 17.8% of the phenotypic variance in RACI-UK, the 10 epistatic pairs jointly explained about 2.5% of the phenotypic variance mainly (i.e. 1.9%) by interactions, suggesting the existence of widespread GxG interactions within MHC that are individually moderate/weak (on average explaining <0.2% of phenotypic variance) but jointly appreciable. The additive background and the 10 epistatic pairs explained 17.1% and 1.7% (0.8% by interactions) of the phenotypic variance in RACI-US, and 14.5% and 1.7% (0.7% by interactions) in RACI-SE.

Combining the three study cohorts together boosted power for both the GWAS and vGWAS analyses as expected (Supplementary Figure S7), with no signs of inflation or deflation (Supplementary Figure S8). The vGWAS analysis identified two new significant loci *ANKRD55* and *TYK2* in addition to MHC and *PTPN22*, which were all genome-wide significant in the GWAS analysis. The top vGWAS SNPs rs71624119 for *ANKRD55* (P = 4.9e-07) and rs34536443 for *TYK2* (P = 7.0e-06) both had only weak or no signals in individual member cohorts, i.e. the P values were 4.4e-03, 2.5e-02 and 7.9e-02 in RACI-UK, RACI-US and RACI-SE respectively for rs71624119; and 1.8e-04, 3.4e-01 and 2.1e-01 respectively for rs34536443. In contrast, the GWAS analysis identified additional 12 genome-wide significant loci that were insignificant in the vGWAS (Supplementary Figure S7).

We plotted the vGWAS P values against the GWAS counterparts for the Combined cohort as well as each individual cohort to assess the impact of the mean-variance correlation (Fig. 3). In the Combined cohort, high concordance between GWAS and vGWAS was observed when the MHC and/or *PTPN22* regions were included (Fig. 3a,b) but greatly diminished if excluding both MHC and *PTPN22* (Fig. 3c). In individual cohorts, GWAS and vGWAS concordance was observed only in RACI-UK even when including *PTPN22* (Fig. 3d,e,f). These results suggested that the mean-variance correlation might influence vGWAS directly mainly at the major GWAS loci. In addition, we reran the vGWAS analyses while fitting the additive effects of the pre-identified GWAS SNPs as additional covariates at either stage in RACI-UK and found little differences in the results, indicating the vGWAS signals were unlikely driven by the differences of allelic means or scaling factors.

## Discussion

We showed that the two-stage vGWAS approach successfully identified MHC that harbored widespread genotypic variability of non-additive risk of RA across the three study cohorts. Such non-additive genotypic variability could be explained in part by GxG interactions across the entire MHC region that were independent of the strong additive effects, and had a moderate/weak effect individually but an appreciable joint effect. We also identified *PTPN22* as a significant vGWAS locus in the discovery cohort that however was replicated in only one independent cohort. Combining the three study cohorts boosted the power of vGWAS and detected two additional loci *ANKRD55* and *TYK2* that are yet to be replicated. These results suggest that the two-stage vGWAS indeed works for complex diseases.

Our results of multiple GxG interactions within MHC are in line with the recent reports that focused on a few preselected MHC loci (e.g. *HLA-DRB1*) and used high resolution imputation and a much increased sample size to fine map within-locus interactions^29^,^39^. Similar fine mapping efforts are needed to accurately compute the additive background and subsequently refine GxG interactions across the entire MHC region. There is not information of environmental factors other than gender in the study cohorts to test GxE interactions. However, there have been several reports of GxE interactions (e.g. with smoking and alcohol) in RA involving MHC and/or *PTPN22*^40^,^41^,^42^,^43^,^44^. Besides, there is also evidence of non-additive effects and heterozygous advantage in MHC variants from other autoimmune diseases^29^,^38^,^39^, all supporting the notion that MHC is highly diverse owing to strong natural selection pressure in order to cope with ever changing environmental pathogens^45^.

One major concern about vGWAS applications is potential conflation caused by the mean-variance correlation that is hardly disentangled, i.e. variance can be mathematically modelled as some function of the mean^20^,^46^. Here we showed that the impact of such a correlation was strong only in the major RA associated loci (i.e. MHC and *PTPN22*) that tended to be involved in GxG and/or GxE interactions. We argue that co-existence of strong additive and non-additive effects in the major associated loci may be biologically important^9^ rendering these loci the key regulators of the study phenotype and even other related phenotypes (e.g. MHC regulates a number of autoimmune diseases). From this perspective, detection of the major GWAS loci should not be regarded as a rediscovery but in fact an assurance that vGWAS can at least pick up the key interacting loci for dichotomous disease phenotypes.

vGWAS may be more fruitful in meta-analysis of multiple cohorts as demonstrated in the Combined cohort analysis. We showed that the newly discovered vGWAS loci, like those in GWAS meta-analysis, had weaker association signals than MHC or *PTPN22*, suggesting that they are unlikely involved in strong interactions and thus require more samples to detect^9^. *ANKRD55* is associated with multiple diseases including RA and multiple sclerosis^3^,^47^ but its biological functions and involvement in statistical interaction are not yet clear. *TYK2* is also a pleiotropic gene associated with multiple autoimmune diseases and was found interacting with *IRF5* in lupus but the interactions seemed weak and inconsistent^48^,^49^,^50^. Further work is required to validate their non-additive roles in RA. We anticipate that future enlarged vGWAS meta-analyses may detect additional, especially novel loci that may be driven more by the variance rather than the mean.

vGWAS is also useful to increase power of detection of interactions via an effective reduction of multiple tests concerning only the prioritized loci. For example, testing GxG interactions benefited from a much relaxed significance threshold as 4.2e-06 after filtering on vGWAS significant SNPs and then their pairwise LD in this study. Caution is recommended when using such filtering because vGWAS could miss true interacting loci with limited genotypic variability^9^,^11^,^12^. Nevertheless, such a filtering is practically useful to prioritize the main non-additive loci particularly when a big sample size is available.

This pilot study has a few limitations. Firstly, the low power of detection in individual cohorts (particularly in RACI-US and RACI-SE). In addition to sample size and SNP density, power of a vGWAS is also determined by the effect sizes of the interactions involved and the unobserved interacting factors^11^,^36^. The MHC signals appeared significantly in every cohort probably because they were involved in multiple interactions as suggested previously^17^. Secondly, uncertainties associated with the estimated environmental residuals due to hidden or unaccounted factors. Factors influencing the accuracy of polygenic risk prediction (e.g. sample size, case control ratio, SNP density, population structure, sample relatedness and ascertainment)^31^,^32^,^33^ will also influence the vGWAS power. Population histories and/or special environments may also cause biases if not unaccounted for properly. For example, RACI-SE was the only cohort using matched cases and controls^2^, which might explain in part the relatively low estimate of heritability (Table 1) and the slight deflation observed in the vGWAS QQ plot (Supplementary Figure S1). Thirdly, this study concerned only the Immunochip platform for an autoimmune disease. Further investigations using GWAS arrays and other types of diseases would be useful to provide a complete view of this approach. A carefully designed simulation study is also recommended to assess the factors above as well as to quantify the impact of the mean-variance correlation comprehensively. Such a simulation study may also help develop a consensus protocol to facilitate future vGWAS meta-analysis of diseases.

## Additional Information

**How to cite this article**: Wei, W.-H. *et al*. Major histocompatibility complex harbors widespread genotypic variability of non-additive risk of rheumatoid arthritis including epistasis. *Sci. Rep.* **6**, 25014; doi: 10.1038/srep25014 (2016).

## Supplementary Material

Supplementary Information

## Figures and Tables

**Table 1:** Study cohorts and vGWAS results*.

cohort	SNPs	cases	controls	heritability (se)	vGWAS
RACI-UK	124 363	2402	8411	0.247 (0.013)	1639
RACI-US	118 550	1800	2125	0.246 (0.017)	154
RACI-SE	123 146	1755	1931	0.185 (0.018)	343

^*^only ACPA+ cases were included in each cohort; heritability (se): polygenic heritability estimate and standard error in brackets.

**Table 2:** The ten independent epistatic pairs of SNPs within MHC and their interaction P values conditioning on the additive background in RACI-UK and without conditioning on the additive background in each of the three study cohorts*.

SNP_1_	Position_1_	SNP_2_	Position_2_	Conditional_P	Unconditional_P_UK	Unconditional_P_US	Unconditional_P_SE
rs805286	31 711 530	rs532098	32 610 275	2.5e-07	1.3e-16	6.9e-04	4.6e-04
rs9266629	31 379 045	rs3830076	32 128 467	1.0e-04	2.2e-14	4.4e-04	1.5e-04
rs9267833	32 210 123	rs2858312	32 699 453	2.5e-03	2.3e-14	2.2e-18	9.9e-12
rs3177928	32 444 658	rs9368737	32 690 652	5.6e-04	7.7e-16	2.9e-02	1.0e-02
rs3997849	32 714 625	rs3892710	32 715 085	3.2e-05	1.9e-38	5.0e-18	3.2e-15
rs9366778	31 301 396	rs2239707	31 557 542	7.1e-04	1.6e-14	8.7e-02	2.6e-03
rs2517448	31 094 890	rs2256594	32 219 095	3.2e-04	3.3e-17	1.3e-05	2.8e-02
rs4947342	32 685 293	rs2857126	32 809 363	1.0e-03	6.5e-13	1.0e-03	2.2e-02
rs241433	32 831 018	rs2239701	32 837 272	7.9e-04	9.5e-24	5.1e-07	6.2e-03
rs2736426	31 777 507	rs11244	32 812 946	6.3e-04	2.0e-16	4.6e-04	2.3e-12

^*^SNP_1(2)_: the first (second) epistatic SNP; Position_1(2)_: the genomic postion of the SNP_1(2)_; Conditional_P: the interaction P value in the final model fitting covariates, the additive effects of the 20 independent GWAS SNPs and the 10 epistatic pairs of SNPs and their interactions; Unconditional_P_UK (US or SE): the interaction P value corrected for covariates but not the additive background in RACI-UK (US or SE); P values greater than 0.05 underlined.

## References

[b1] Diogo D., Okada Y. & Plenge R. M. Genome-wide association studies to advance our understanding of critical cell types and pathways in rheumatoid arthritis: recent findings and challenges. Curr Opin Rheumatol 26, 85–92 (2014). 24276088 10.1097/BOR.0000000000000012

[b2] Eyre S. . High-density genetic mapping identifies new susceptibility loci for rheumatoid arthritis. Nat Genet 44, 1336–1340 (2012). 23143596 10.1038/ng.2462 PMC3605761

[b3] Okada Y. . Genetics of rheumatoid arthritis contributes to biology and drug discovery. Nature 506, 376–381 (2014). 24390342 10.1038/nature12873 PMC3944098

[b4] Viatte S., Plant D. & Raychaudhuri S. Genetics and epigenetics of rheumatoid arthritis. Nat Rev Rheumatol 9, 141–153 (2013). 23381558 10.1038/nrrheum.2012.237 PMC3694322

[b5] Parkes M., Cortes A., van Heel D. A. & Brown M. A. Genetic insights into common pathways and complex relationships among immune-mediated diseases. Nat Rev Genet 14, 661–673 (2013). 23917628 10.1038/nrg3502

[b6] Zuk O., Hechter E., Sunyaev S. R. & Lander E. S. The mystery of missing heritability: Genetic interactions create phantom heritability. Proc Natl Acad Sci USA 109, 1193–1198 (2012). 22223662 10.1073/pnas.1119675109 PMC3268279

[b7] Thomas D. Gene–environment-wide association studies: emerging approaches. Nat Rev Genet 11, 259–272 (2010). 20212493 10.1038/nrg2764 PMC2891422

[b8] Lehner B. Genotype to phenotype: lessons from model organisms for human genetics. Nat Rev Genet 14, 168–178 (2013). 23358379 10.1038/nrg3404

[b9] Wei W.-H., Hemani G. & Haley C. S. Detecting epistasis in human complex traits. Nat Rev Genet 15, 722–733 (2014). 25200660 10.1038/nrg3747

[b10] Robinson M. R., Wray N. R. & Visscher P. M. Explaining additional genetic variation in complex traits. Trends Genet 30, 124–132 (2014). 24629526 10.1016/j.tig.2014.02.003 PMC4639398

[b11] Struchalin M. V., Dehghan A., Witteman J. C., van Duijn C. & Aulchenko Y. S. Variance heterogeneity analysis for detection of potentially interacting genetic loci: method and its limitations. BMC Genet 11, 92 (2010). 20942902 10.1186/1471-2156-11-92 PMC2973850

[b12] Ronnegard L. & Valdar W. Recent developments in statistical methods for detecting genetic loci affecting phenotypic variability. BMC Genet 13, 63 (2012). 22827487 10.1186/1471-2156-13-63 PMC3493319

[b13] Shen X., Pettersson M., Rönnegård L. & Carlborg Ö. Inheritance Beyond Plain Heritability: Variance-Controlling Genes in *Arabidopsis thaliana*. PLos Genet 8, e1002839 (2012). 22876191 10.1371/journal.pgen.1002839 PMC3410891

[b14] Ayroles J. F. . Behavioral idiosyncrasy reveals genetic control of phenotypic variability. Proc Natl Acad Sci USA 112, 6706–6711 (2015). 25953335 10.1073/pnas.1503830112 PMC4450409

[b15] Falconer D. S. Selection in different environments: effects on environmental sensitivity (reaction norm) and on mean performance. Genet Res (Camb) 56, 57–70 (1990).

[b16] Visscher P. M. & Posthuma D. Statistical power to detect genetic Loci affecting environmental sensitivity. Behav Genet 40, 728–733 (2010). 20428936 10.1007/s10519-010-9362-0

[b17] Brown A. A. . Genetic interactions affecting human gene expression identified by variance association mapping. Elife 3, e01381 (2014). 24771767 10.7554/eLife.01381 PMC4017648

[b18] Pare G., Cook N. R., Ridker P. M. & Chasman D. I. On the use of variance per genotype as a tool to identify quantitative trait interaction effects: a report from the Women’s Genome Health Study. PLos Genet 6, e1000981 (2010). 20585554 10.1371/journal.pgen.1000981 PMC2887471

[b19] Yang J. . FTO genotype is associated with phenotypic variability of body mass index. Nature 490, 267–272 (2012). 22982992 10.1038/nature11401 PMC3564953

[b20] Sun X., Elston R., Morris N. & Zhu X. What Is the Significance of Difference in Phenotypic Variability across SNP Genotypes? Am J Hum Genet 93, 390–397 (2013). 23910463 10.1016/j.ajhg.2013.06.017 PMC3738833

[b21] Hoggart C. J. . Novel Approach Identifies SNPs in *SLC2A10* and *KCNK9* with Evidence for Parent-of-Origin Effect on Body Mass Index. PLos Genet 10, e1004508 (2014). 25078964 10.1371/journal.pgen.1004508 PMC4117451

[b22] Topless R. . Association of *SLC2A9* genotype with phenotypic variability of serum urate in pre-menopausal women. Front Genet 6, 313 (2015). 26528330 10.3389/fgene.2015.00313 PMC4604317

[b23] Hulse A. M. & Cai J. J. Genetic variants contribute to gene expression variability in humans. Genetics 193, 95–108 (2013). 23150607 10.1534/genetics.112.146779 PMC3527258

[b24] Wang G., Yang E., Brinkmeyer-Langford C. L. & Cai J. J. Additive, epistatic, and environmental effects through the lens of expression variability QTL in a twin cohort. Genetics 196, 413–425 (2014). 24298061 10.1534/genetics.113.157503 PMC3914615

[b25] Franks P. W., Pearson E. & Florez J. C. Gene-environment and gene-treatment interactions in type 2 diabetes: progress, pitfalls, and prospects. Diabetes Care 36, 1413–1421 (2013). 23613601 10.2337/dc12-2211 PMC3631878

[b26] Claussnitzer M. . FTO Obesity Variant Circuitry and Adipocyte Browning in Humans. N Engl J Med 373, 895–907 (2015). 26287746 10.1056/NEJMoa1502214 PMC4959911

[b27] Dominguez-Reyes T. . Interaction of dietary fat intake with APOA2, APOA5 and LEPR polymorphisms and its relationship with obesity and dyslipidemia in young subjects. Lipids Health Dis 14, 106 (2015). 26365669 10.1186/s12944-015-0112-4 PMC4568066

[b28] Wei W. H. . Abundant local interactions in the 4p16.1 region suggest functional mechanisms underlying SLC2A9 associations with human serum uric acid. Hum Mol Genet 23, 5061–5068 (2014). 24821702 10.1093/hmg/ddu227 PMC4159153

[b29] Lenz T. L. . Widespread non-additive and interaction effects within HLA loci modulate the risk of autoimmune diseases. Nat Genet 47, 1085–1090 (2015). 26258845 10.1038/ng.3379 PMC4552599

[b30] Yang J., Lee S. H., Goddard M. E. & Visscher P. M. GCTA: a tool for genome-wide complex trait analysis. Am J Hum Genet 88, 76–82 (2011). 21167468 10.1016/j.ajhg.2010.11.011 PMC3014363

[b31] Lee S. H. . Estimating the proportion of variation in susceptibility to schizophrenia captured by common SNPs. Nat Genet 44, 247–250 (2012). 22344220 10.1038/ng.1108 PMC3327879

[b32] Lee S. H., Wray N. R., Goddard M. E. & Visscher P. M. Estimating missing heritability for disease from genome-wide association studies. Am J Hum Genet 88, 294–305 (2011). 21376301 10.1016/j.ajhg.2011.02.002 PMC3059431

[b33] Chen G. B. . Estimation and partitioning of (co)heritability of inflammatory bowel disease from GWAS and immunochip data. Hum Mol Genet 23, 4710–4720 (2014). 24728037 10.1093/hmg/ddu174 PMC4119411

[b34] Cortes A. & Brown M. A. Promise and pitfalls of the Immunochip. Arthritis Res Ther 13, 101 (2011). 21345260 10.1186/ar3204 PMC3157635

[b35] Purcell S. . PLINK: a tool set for whole-genome association and population-based linkage analyses. Am J Hum Genet 81, 559–575 (2007). 17701901 10.1086/519795 PMC1950838

[b36] Struchalin M. V., Amin N., Eilers P. H., van Duijn C. M. & Aulchenko Y. S. An R package “VariABEL” for genome-wide searching of potentially interacting loci by testing genotypic variance heterogeneity. BMC Genet 13, 4 (2012). 22272569 10.1186/1471-2156-13-4 PMC3398297

[b37] Aulchenko Y. S., Ripke S., Isaacs A. & van Duijn C. M. GenABEL: an R library for genome-wide association analysis. Bioinformatics 23, 1294–1296 (2007). 17384015 10.1093/bioinformatics/btm108

[b38] Goyette P. . High-density mapping of the MHC identifies a shared role for HLA-DRB1*01:03 in inflammatory bowel diseases and heterozygous advantage in ulcerative colitis. Nat Genet 47, 172–179 (2015). 25559196 10.1038/ng.3176 PMC4310771

[b39] Hu X. . Additive and interaction effects at three amino acid positions in HLA-DQ and HLA-DR molecules drive type 1 diabetes risk. Nat Genet 47, 898–905 (2015). 26168013 10.1038/ng.3353 PMC4930791

[b40] Kallberg H. . Gene-gene and gene-environment interactions involving HLA-DRB1, PTPN22, and smoking in two subsets of rheumatoid arthritis. Am J Hum Genet 80, 867–875 (2007). 17436241 10.1086/516736 PMC1852748

[b41] Karlson E. W. . Gene-environment interaction between HLA-DRB1 shared epitope and heavy cigarette smoking in predicting incident rheumatoid arthritis. Ann Rheum Dis 69, 54–60 (2010). 19151010 10.1136/ard.2008.102962 PMC2952498

[b42] Lu B. . Associations of smoking and alcohol consumption with disease activity and functional status in rheumatoid arthritis. J Rheumatol 41, 24–30 (2014). 24293566 10.3899/jrheum.130074 PMC4017580

[b43] Morgan A. W. . Reevaluation of the interaction between HLA-DRB1 shared epitope alleles, PTPN22, and smoking in determining susceptibility to autoantibody-positive and autoantibody-negative rheumatoid arthritis in a large UK Caucasian population. Arthritis Rheum 60, 2565–2576 (2009). 19714585 10.1002/art.24752

[b44] Kim K. . Interactions between amino-acid-defined MHC class II variants and smoking for seropositive rheumatoid arthritis. Arthritis Rheumatol 67, 2611–2623 (2015). 26098791 10.1002/art.39228 PMC4581918

[b45] Horton R. . Gene map of the extended human MHC. Nat Rev Genet 5, 889–899 (2004). 15573121 10.1038/nrg1489

[b46] Shen X. & Ronnegard L. Issues with data transformation in genome-wide association studies for phenotypic variability. F1000Res 2, 200 (2013). 24555098 10.12688/f1000research.2-200.v1 PMC3869493

[b47] Alloza I. . ANKRD55 and DHCR7 are novel multiple sclerosis risk loci. Genes Immun 13, 253–257 (2012). 22130326 10.1038/gene.2011.81

[b48] Hellquist A. . Evidence for genetic association and interaction between the TYK2 and IRF5 genes in systemic lupus erythematosus. J Rheumatol 36, 1631–1638 (2009). 19567624 10.3899/jrheum.081160

[b49] Suarez-Gestal M., Calaza M. & Gonzalez A. Lack of interaction between systemic lupus erythematosus-associated polymorphisms in TYK2 and IRF5. J Rheumatol 37, 676–677; author reply 678 (2010). 20197570 10.3899/jrheum.090823

[b50] Tang L. . Genetic association and interaction between the IRF5 and TYK2 genes and systemic lupus erythematosus in the Han Chinese population. Inflamm Res 64, 817–824 (2015). 26294277 10.1007/s00011-015-0865-2

