# Supplementary material for: Major histocompatibility complex harbors widespread genotypic variability of non-additive risk of rheumatoid arthritis including epistasis
Source: Sci Rep. 2016 Apr 25;6:25014. doi: 10.1038/srep25014 (PMC4842957; doi:10.1038/srep25014)
Supplement: Supplementary Information [file srep25014-s1.pdf]

Major histocompatibility complex harbors widespread genotypic variability of non-additive risk of rheumatoid arthritis including epistasis

Wen-Hua Wei,[1,2,*] John Bowes,[1] Darren Plant,[1] Sebastien Viatte,[1] Annie Yarwood,[1] Jonathan Massey,[1] Jane Worthington,[1,3] Stephen Eyre[1,3]

[1]Arthritis Research UK Centre for Genetics and Genomics, Institute of Inflammation and Repair, Faculty of Medical and Human Sciences, Manchester Academic Health Science Centre, University of Manchester, Oxford Road, Manchester M13 9PT, UK

[2]Department of Women's and Children's Health, Dunedin School of Medicine, University of Otago, Dunedin 9016, New Zealand

[3]NIHR Manchester Musculoskeletal Biomedical Research Unit, Central Manchester NHS Foundation Trust, Manchester Academic Health Science Centre, Manchester, UK

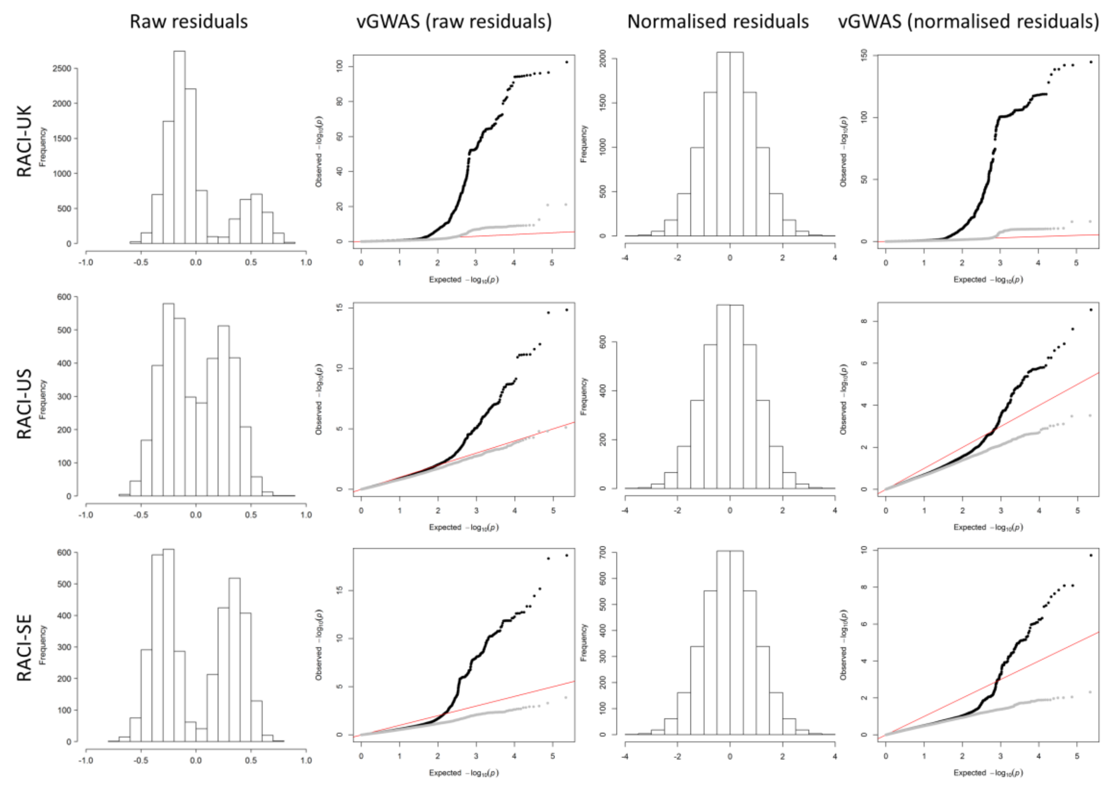

Supplementary Figure S1: Histograms of the environmental residuals (raw or normalized) and quantile-quantile plots of corresponding vGWAS analyses in RACI-UK (top row), RACI-US (middle row) and RACI-SE (bottom row). Series excluding MHC are in grey in QQ plots

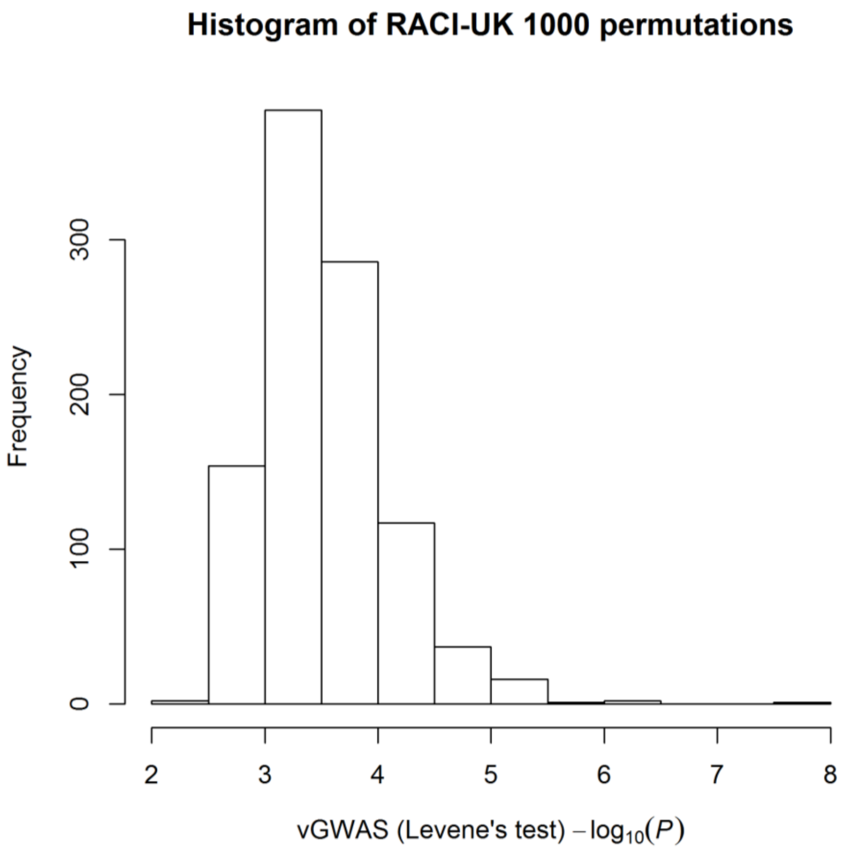

Supplementary Figure S2: Histograms of the lowest P values (at the –log10 scale) in each of the 1000 vGWASs based on randomly permuted environmental residuals in the RACI-UK cohort

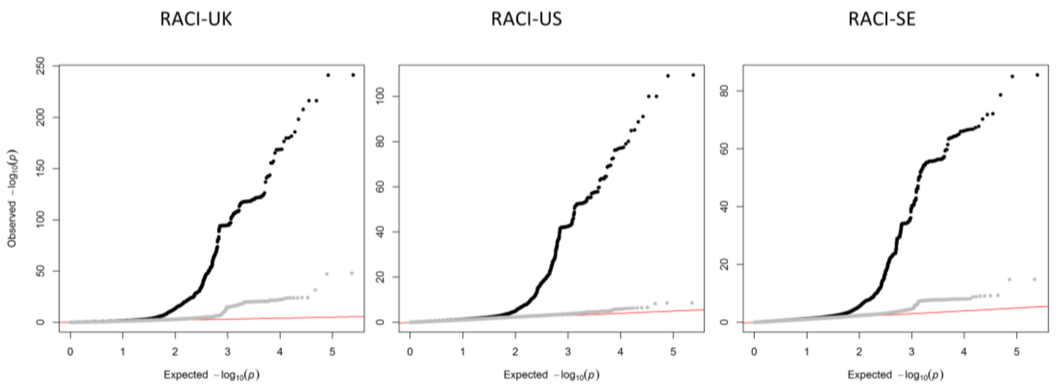

Supplementary Figure S3: Quantile-quantile plots of the conventional GWAS analyses (series excluding MHC are in grey) in RACI-UK, RACI-US and RACI-SE

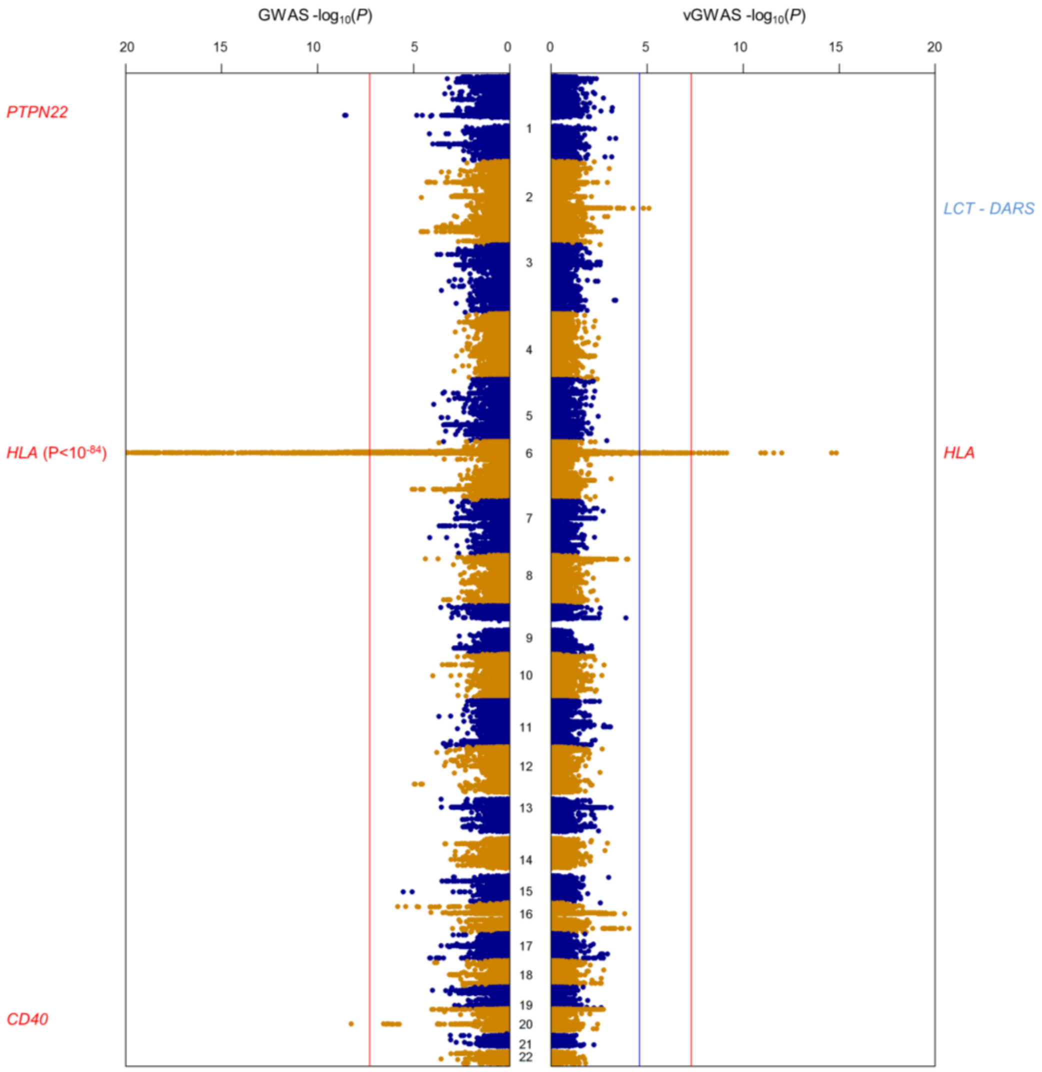

Supplementary Figure S4: Aligned Manhattan plots of GWAS (left) and vGWAS (right) analyses in RACI-US. P values are at the -$\log_{10}$ scale; red line represents GWAS genome-wide significance threshold; blue line represents vGWAS significance threshold derived from permutation; significant loci are annotated to genes in red (or blue if reached only the vGWAS significance threshold)

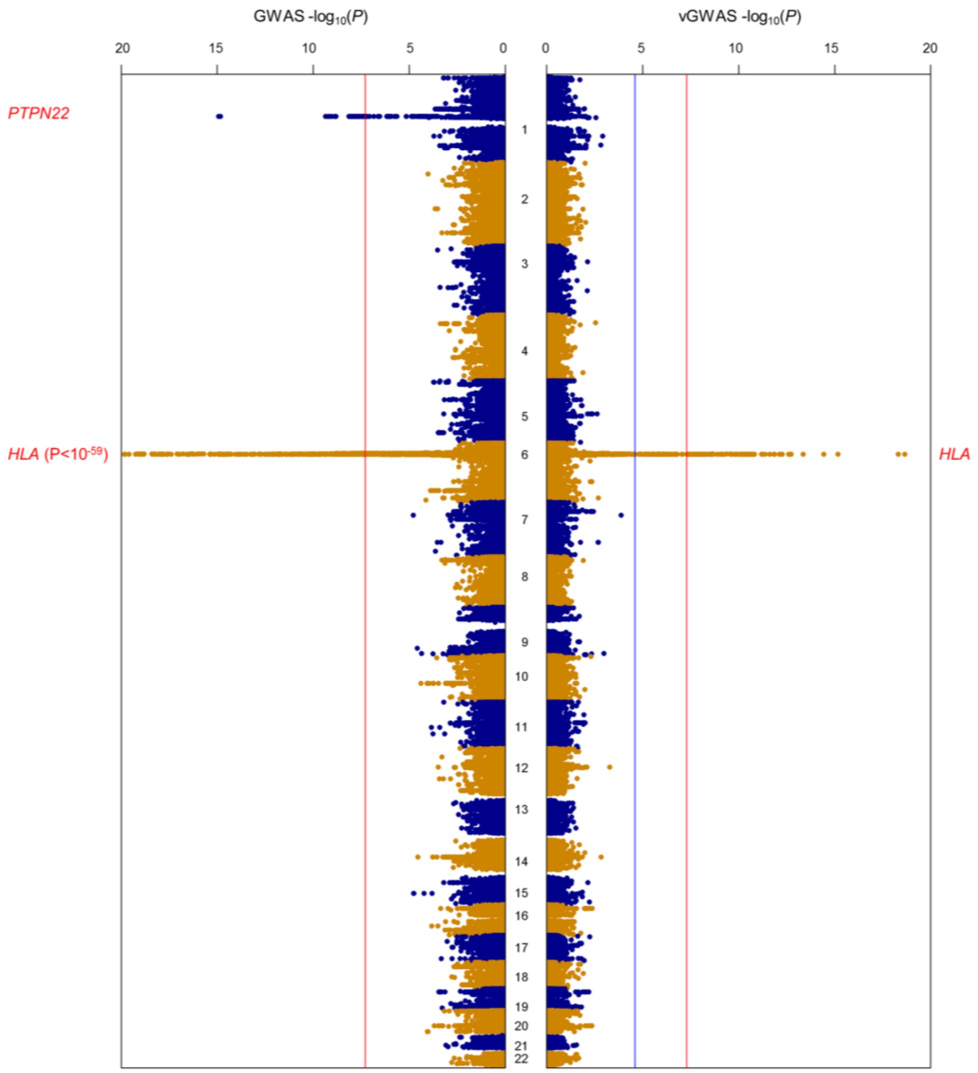

Supplementary Figure S5: Aligned Manhattan plots of GWAS (left) and vGWAS (right) analyses in RACI-SE. P values are at the $-\log_{10}$ scale; red line represents GWAS genome-wide significance threshold; blue line represents vGWAS significance threshold derived from permutation; significant loci are annotated to genes in red (or blue if reached only the vGWAS significance threshold)

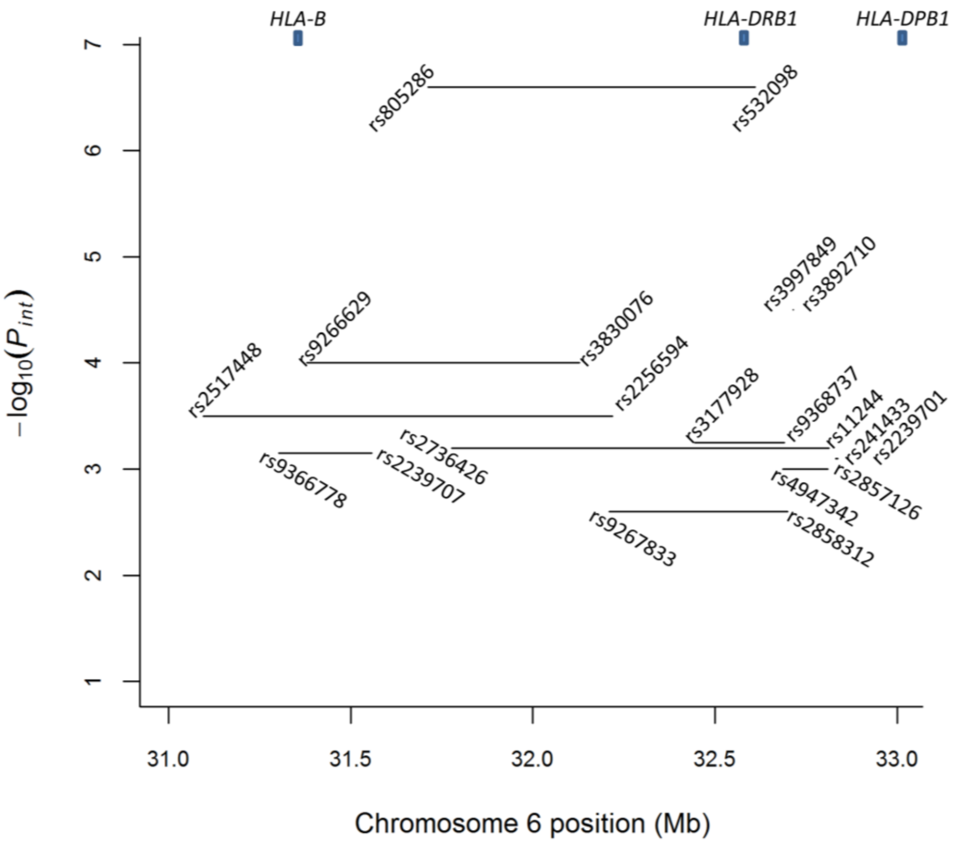

Supplementary Figure S6: Alignment of ten independent epistatic pairs of SNPs within the MHC region in RACI-UK. Each horizontal line represents an interaction between a start SNP (left) and an end SNP (right) (lines very short for rs3997849 - rs3892710 and rs241433 - rs2239701); each dark small bar and a gene label at the top of graph represents a major RA gene within MHC; y-axis: conditional interaction P-values in the -log10 scale; x-axis: genomic location in megabase (GRCh38/hg38)

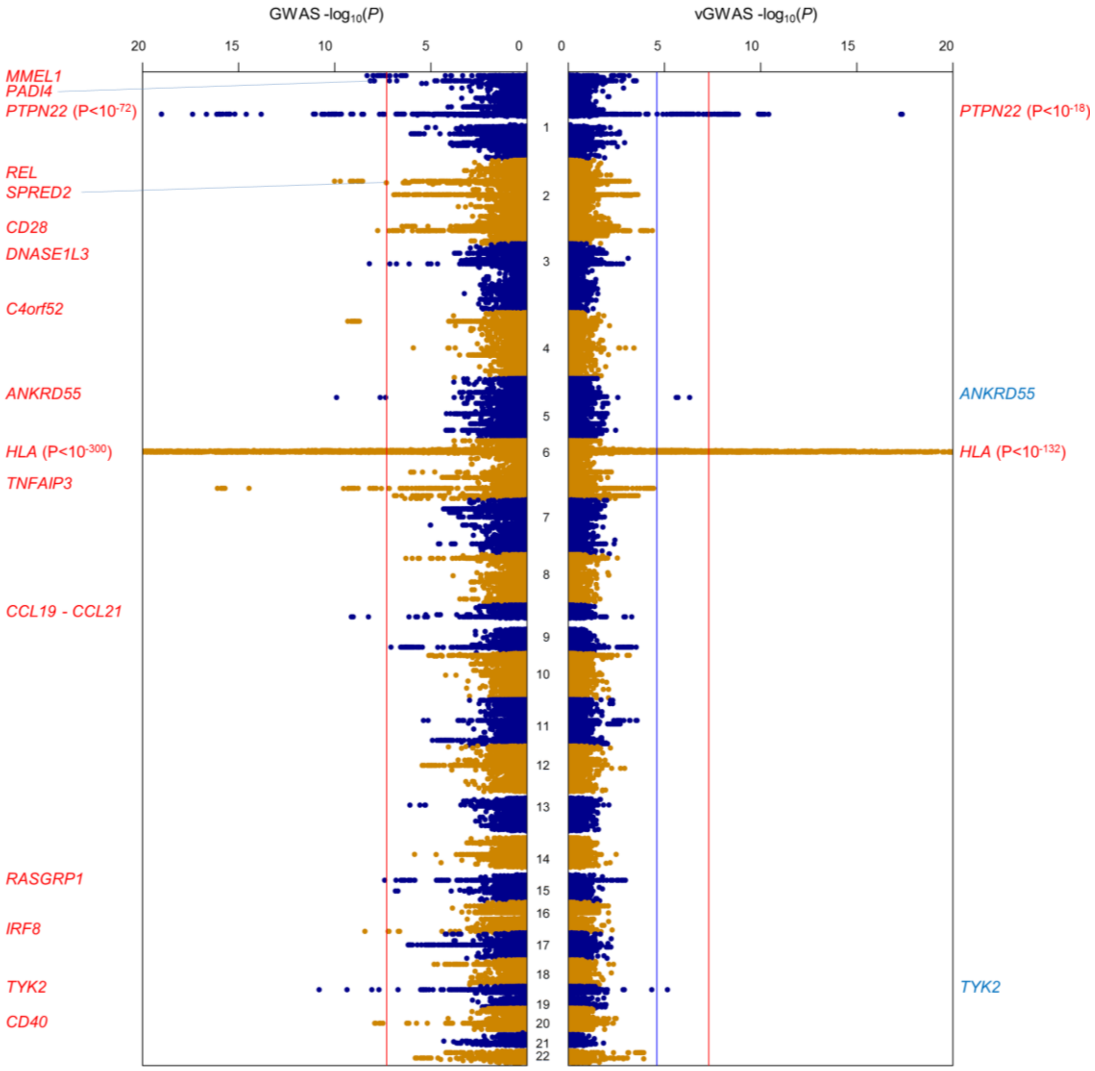

Supplementary Figure S7: Aligned Manhattan plots of GWAS (left) and vGWAS (right) analyses in the cohort combined RACI-UK, RACI-US and RACI-SE. P values are at the $-\log_{10}$ scale; red line represents GWAS genome-wide significance threshold; blue line represents vGWAS significance threshold derived from permutation; significant loci are annotated to genes in red (or blue if reached only the vGWAS significance threshold)

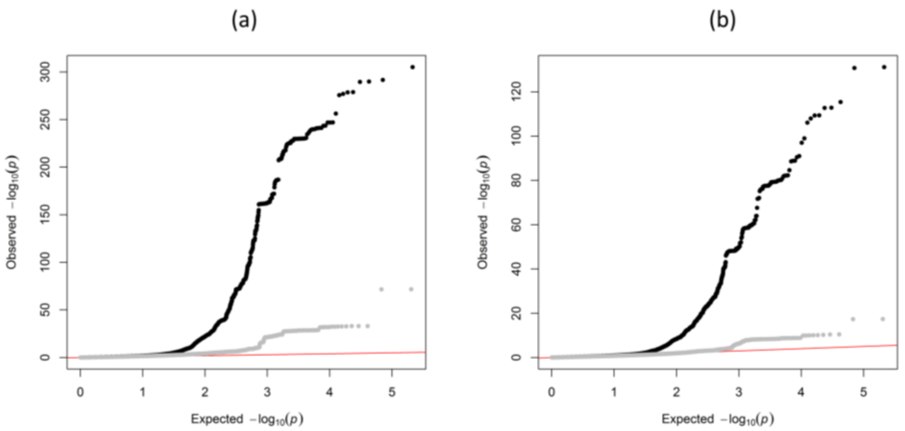

Supplementary Figure S8: Quantile-quantile plots of the GWAS and vGWAS analyses in the Combined cohort. (a) GWAS QQ plot with and without MHC (grey); (b) vGWAS QQ plot with and without MHC (grey)

Supplementary Table S1: Forward selection choose 20 independent GWAS significant SNPs in RACI-UK

SNP	Chromosome	Position	GWAS_P_value
rs6931277	6	32 615 580	4.23E-242
rs17496549	6	32 441 931	6.61E-14
rs2476601	1	113 834 946	1.36E-48
rs2596560	6	31 387 541	2.15E-09
rs9277538	6	33 087 270	4.72E-20
rs2269425	6	32 155 862	5.71E-11
rs928722	6	137 652 695	1.70E-10
rs34536443	19	10 352 442	4.65E-09
rs3819721	6	32 837 021	4.70E-84
rs1233394	6	29 581 484	2.83E-10
rs4540292	6	31 349 405	1.39E-13
rs17422797	6	32 297 751	1.40E-15
rs9268543	6	32 417 024	1.63E-208
rs13196329	6	32 357 594	7.91E-10
rs10484565	6	32 827 255	2.65E-25
rs384247	6	32 216 797	3.90E-13
rs2524084	6	31 273 862	1.06E-15
rs2242656	6	31 646 325	5.74E-12
rs3104407	6	32 714 675	1.96E-41
rs2097431	6	32 623 056	9.29E-11

Supplementary Table S2: The directly replicated MHC SNPs and their P values in vGWAS and GWAS in three cohorts

SNP	Position	RACI-UK_vGWAS	RACI-UK_GWAS	RACI-US_vGWAS	RACI-US_GWAS	RACI-SE_vGWAS	RACI-SE_GWAS
rs2255221	31 463 914	9.55E-07	7.43E-12	4.07E-03	1.37E-08	1.04E-03	1.44E-05
rs2857694	31 620 093	9.71E-15	1.11E-28	6.17E-03	1.36E-08	9.02E-03	5.43E-17
rs2736171	31 627 710	4.08E-15	1.05E-29	4.01E-03	2.96E-08	9.16E-03	3.97E-17
rs2261033	31 635 814	4.31E-39	1.06E-67	1.52E-03	1.58E-27	1.69E-03	1.14E-24
rs28366155	31 690 857	5.61E-11	6.07E-19	4.91E-03	1.67E-11	1.10E-02	7.35E-05
rs6916278	31 710 997	4.80E-11	4.84E-19	8.99E-03	9.04E-11	9.73E-03	5.60E-05
rs805284	31 714 252	1.12E-31	1.46E-60	3.01E-04	1.46E-25	5.48E-03	1.55E-18
rs707939	31 758 911	3.30E-33	6.01E-62	1.24E-02	4.76E-21	1.20E-04	2.48E-22
rs2075800	31 810 169	8.59E-35	4.37E-65	1.15E-02	2.55E-21	2.53E-04	9.27E-23
rs521977	31 869 050	9.59E-14	4.82E-22	1.12E-03	6.01E-09	3.27E-02	1.14E-10
rs494620	31 870 936	8.28E-24	1.11E-43	2.69E-02	1.73E-17	6.17E-05	3.28E-22
rs614549	31 872 848	1.77E-27	1.26E-50	4.16E-02	1.45E-18	1.34E-04	4.60E-24
rs2736428	31 876 147	6.10E-30	3.33E-55	3.79E-02	3.54E-18	5.60E-04	8.13E-20
rs6907185	31 876 907	1.04E-08	6.14E-16	1.10E-03	2.70E-11	6.76E-06	2.01E-07
rs2844458	31 883 692	1.47E-30	1.29E-56	1.96E-02	1.56E-16	6.73E-04	1.41E-19
rs535586	31 892 560	1.84E-14	2.19E-24	9.44E-04	1.01E-07	1.77E-02	8.65E-11
rs644045	31 916 180	4.22E-22	1.21E-37	1.40E-02	1.41E-13	1.50E-02	1.13E-15
rs544167	31 922 381	1.38E-09	5.06E-17	1.45E-02	7.50E-10	2.77E-10	2.72E-10
rs2734335	31 926 167	1.15E-26	2.78E-47	1.20E-02	7.98E-17	1.26E-02	4.92E-17
rs3020644	31 926 849	1.98E-24	9.79E-45	4.37E-02	5.27E-11	1.75E-02	3.33E-10
rs621701	31 935 344	1.58E-09	5.96E-17	1.21E-02	2.96E-10	4.27E-10	3.27E-10
rs550605	31 939 370	4.05E-11	1.08E-20	1.13E-02	3.91E-11	2.20E-09	3.88E-11
rs541862	31 949 174	2.40E-11	3.78E-21	1.15E-02	9.04E-11	2.55E-09	5.74E-11
rs4151657	31 949 763	3.31E-33	6.38E-61	3.42E-02	1.25E-17	1.30E-03	8.18E-18
rs2072633	31 951 801	1.23E-29	3.41E-51	2.79E-03	9.57E-21	5.12E-03	7.57E-25

rs760070	31 952 179	2.54E-11	4.82E-21	1.11E-02	6.14E-11	2.55E-09	5.74E-11
rs592229	31 962 664	8.63E-21	9.51E-37	4.45E-03	1.81E-10	3.64E-03	1.45E-12
rs444921	31 964 400	2.92E-10	9.86E-19	4.97E-03	3.44E-09	1.56E-04	1.97E-06
rs406936	31 965 384	2.64E-10	7.66E-19	4.28E-03	1.01E-09	1.60E-04	2.17E-06
rs454212	31 966 595	2.60E-10	7.64E-19	4.60E-03	2.00E-09	1.51E-04	1.91E-06
rs449643	31 968 902	2.60E-10	7.64E-19	4.71E-03	1.56E-09	1.49E-04	1.91E-06
rs387608	31 973 780	2.30E-10	5.01E-19	4.75E-03	2.42E-09	1.31E-04	1.27E-06
rs389512	31 979 817	6.22E-10	2.43E-18	9.79E-03	5.64E-09	2.01E-04	3.56E-06
rs2857009	32 051 969	1.39E-36	5.15E-67	2.76E-02	5.06E-19	1.53E-04	5.42E-21
rs6902493	32 055 121	1.00E-36	2.63E-67	2.43E-02	1.41E-18	2.60E-04	2.98E-21
rs2239689	32 062 507	1.02E-36	3.76E-67	2.66E-02	1.95E-18	3.11E-04	2.85E-21
rs7766862	32 065 230	6.81E-37	2.03E-67	2.18E-02	1.79E-18	2.20E-04	1.12E-21
rs2071295	32 070 923	1.49E-36	1.01E-66	2.48E-02	1.09E-18	1.86E-04	1.68E-21
rs2071293	32 094 910	2.55E-36	1.81E-67	2.38E-02	1.05E-18	1.35E-04	4.49E-22
rs17421624	32 098 400	4.01E-36	3.26E-67	2.19E-02	5.07E-19	7.94E-05	3.27E-22
rs2269426	32 108 722	2.89E-09	1.47E-17	7.43E-03	7.50E-05	1.75E-02	9.71E-12
rs8111	32 115 398	4.44E-23	1.41E-44	4.09E-02	4.76E-13	4.06E-03	4.49E-16
rs2228628	32 121 077	1.88E-23	9.57E-45	4.71E-02	2.70E-12	4.20E-03	1.48E-16
rs9267803	32 133 985	8.67E-24	3.58E-46	2.70E-02	4.67E-13	3.82E-03	1.66E-17
rs4713505	32 137 224	3.10E-24	6.89E-46	3.44E-02	7.03E-12	4.53E-03	9.64E-17
rs4713506	32 146 203	8.28E-24	1.71E-46	4.03E-02	4.40E-11	9.86E-03	2.02E-16
rs9296009	32 146 738	2.47E-43	2.37E-79	1.62E-02	1.89E-40	4.06E-02	2.77E-21
rs10947233	32 156 647	1.93E-35	1.13E-68	2.09E-03	1.20E-25	4.10E-04	1.71E-22
rs2070600	32 183 666	3.55E-36	2.81E-69	3.22E-03	2.25E-26	7.26E-04	2.61E-23
rs1800684	32 184 217	3.92E-10	4.39E-17	1.07E-02	7.52E-07	4.90E-02	1.24E-04
rs2022059	32 188 712	2.04E-38	1.87E-72	3.61E-03	1.42E-23	1.61E-03	3.39E-21
rs8192575	32 198 607	7.80E-35	7.53E-65	1.96E-03	4.76E-22	8.04E-03	5.57E-19
rs2071285	32 212 654	5.90E-34	6.58E-64	1.92E-03	4.47E-22	8.20E-03	5.30E-19

rs2854050	32 217 828	2.28E-34	1.85E-64	1.91E-03	2.69E-22	7.41E-03	3.86E-19
rs375244	32 223 680	1.76E-10	3.37E-19	8.30E-03	1.44E-11	2.62E-02	8.59E-10
rs396960	32 223 804	5.33E-12	5.93E-22	3.86E-02	2.69E-10	2.01E-02	2.01E-06
rs3134929	32 224 330	4.57E-24	1.62E-43	5.58E-03	7.16E-23	3.05E-08	1.66E-21
rs3096702	32 224 554	1.67E-22	2.37E-42	2.94E-07	9.98E-25	3.72E-04	1.00E-16
rs397081	32 224 840	1.54E-06	7.40E-12	7.69E-04	1.42E-08	4.93E-08	8.34E-11
rs479536	32 225 901	2.86E-09	9.31E-16	4.71E-03	3.55E-09	2.24E-03	1.52E-05
rs9267853	32 227 474	1.44E-22	1.70E-42	3.81E-07	6.78E-24	3.55E-04	1.02E-16
rs6457499	32 231 171	1.03E-18	9.53E-35	3.29E-09	2.37E-26	7.05E-03	1.42E-13
rs9267873	32 231 575	2.19E-25	9.29E-47	2.12E-07	2.88E-24	1.67E-04	6.43E-17
rs9267911	32 237 333	2.19E-26	5.28E-47	1.52E-07	4.65E-26	4.29E-03	8.38E-19
rs6906128	32 238 527	4.91E-16	2.52E-30	9.38E-09	5.75E-25	5.87E-03	4.12E-13
rs416352	32 239 616	6.53E-20	8.71E-36	5.73E-04	6.25E-18	6.56E-03	5.96E-19
rs419132	32 243 022	6.01E-16	2.57E-31	4.73E-07	1.20E-16	1.06E-02	2.22E-11
rs412657	32 243 308	2.10E-29	8.81E-53	1.75E-03	8.83E-27	1.21E-04	5.57E-26
rs9267947	32 243 441	1.96E-22	2.70E-41	2.47E-03	3.90E-21	6.15E-04	1.28E-18
rs9267948	32 244 456	3.10E-36	9.95E-66	4.35E-07	1.43E-30	8.67E-05	3.56E-24
rs17576984	32 245 208	2.13E-07	3.12E-14	4.65E-02	1.73E-07	2.68E-09	1.75E-15
rs9267954	32 245 275	8.28E-57	6.31E-102	6.03E-04	8.28E-43	1.55E-06	7.78E-33
rs9267955	32 245 373	1.91E-15	1.86E-25	8.32E-03	1.05E-10	1.66E-02	2.37E-06
rs454748	32 245 433	3.75E-27	1.73E-47	1.53E-03	7.57E-20	1.26E-03	2.30E-21
rs9267956	32 245 861	6.52E-38	6.53E-69	8.99E-04	1.53E-30	7.64E-06	8.61E-26
rs440169	32 246 011	1.88E-28	1.64E-51	3.78E-03	2.86E-21	4.47E-04	2.29E-23
rs9267958	32 246 839	1.61E-28	1.20E-51	3.71E-03	3.48E-21	4.32E-04	2.38E-23
rs563412	32 247 286	8.85E-29	7.23E-52	5.55E-03	1.64E-21	5.31E-04	7.11E-24
rs405875	32 247 411	6.85E-29	4.79E-52	6.01E-03	1.05E-21	4.19E-04	6.01E-24
rs1559876	32 247 992	1.53E-37	1.82E-68	9.42E-04	2.24E-29	1.20E-05	1.04E-25
rs2894239	32 248 019	1.61E-28	1.20E-51	4.79E-03	1.19E-20	4.39E-04	2.66E-23

rs2395110	32 248 099	6.85E-29	4.79E-52	6.01E-03	1.05E-21	4.19E-04	6.01E-24
rs3115576	32 249 073	1.63E-28	1.19E-51	3.71E-03	3.48E-21	3.91E-04	1.85E-23
rs6457508	32 249 186	1.61E-28	1.20E-51	3.61E-03	3.80E-21	4.86E-04	2.40E-23
rs6457509	32 249 241	3.48E-28	3.70E-51	3.69E-03	2.49E-21	4.05E-04	1.37E-23
rs6936204	32 249 315	7.53E-35	3.84E-58	8.77E-04	2.56E-28	7.28E-05	3.14E-29
rs9267970	32 249 383	1.72E-28	1.32E-51	3.71E-03	3.48E-21	3.96E-04	1.79E-23
rs2894240	32 250 069	1.61E-28	1.20E-51	3.71E-03	3.48E-21	4.23E-04	2.69E-23
rs3115573	32 251 066	1.61E-28	1.20E-51	3.71E-03	3.48E-21	4.11E-04	2.24E-23
rs9267992	32 252 620	2.80E-15	3.21E-25	8.87E-03	9.91E-11	1.65E-02	1.97E-06
rs3115572	32 252 707	5.45E-26	3.08E-43	5.58E-03	2.19E-19	1.40E-04	8.91E-23
rs3130316	32 253 451	4.92E-26	2.28E-43	5.32E-03	2.92E-19	1.37E-04	1.08E-22
rs3115570	32 254 929	1.53E-28	2.25E-51	2.49E-03	2.14E-21	4.92E-04	1.47E-22
rs3130320	32 255 481	8.81E-40	1.09E-65	1.07E-03	6.97E-30	4.42E-06	4.02E-33
rs9268103	32 277 593	9.98E-15	1.79E-26	6.47E-03	4.68E-11	4.49E-02	1.04E-07
rs9268132	32 286 877	5.13E-53	6.05E-95	1.79E-05	3.23E-43	1.11E-06	4.15E-35
rs9268145	32 289 507	3.62E-95	3.75E-169	8.30E-07	2.36E-77	2.80E-11	7.73E-67
rs4713518	32 289 560	5.83E-53	6.11E-95	2.48E-05	1.44E-42	1.02E-06	7.43E-35
rs9268148	32 291 750	1.03E-14	1.85E-26	6.46E-03	4.73E-11	4.92E-02	4.71E-08
rs482194	32 292 782	4.86E-53	5.36E-95	1.88E-05	3.97E-43	1.35E-06	6.21E-35
rs7775397	32 293 475	9.68E-07	1.60E-10	4.24E-02	1.81E-06	8.38E-05	1.44E-09
rs560505	32 293 994	1.91E-54	1.76E-97	1.35E-05	9.89E-43	5.12E-07	1.15E-36
rs28366174	32 298 713	4.30E-10	8.69E-18	2.58E-04	1.40E-09	9.71E-05	4.39E-07
rs537757	32 300 724	4.49E-53	4.63E-95	1.27E-05	2.00E-43	1.11E-06	4.25E-35
rs477005	32 302 723	2.09E-54	2.00E-97	8.22E-06	7.08E-43	4.36E-07	8.17E-37
rs3864299	32 303 897	5.36E-18	2.33E-29	4.94E-02	7.00E-16	9.38E-07	3.84E-23
rs9268177	32 307 105	1.23E-06	2.59E-10	4.28E-02	1.87E-06	8.38E-05	1.44E-09
rs502626	32 310 489	3.24E-53	3.10E-95	2.26E-05	8.38E-43	1.24E-06	5.51E-35
rs9268199	32 310 858	2.32E-20	1.09E-34	2.01E-02	3.71E-17	1.43E-03	5.28E-15

rs6939410	32 312 405	5.64E-19	8.87E-31	4.65E-02	5.45E-18	1.37E-06	1.50E-23
rs1018433	32 313 733	4.03E-18	1.51E-29	3.95E-02	3.84E-16	8.38E-07	5.70E-23
rs547261	32 314 256	2.72E-53	2.39E-95	4.18E-05	1.49E-43	1.21E-06	5.21E-35
rs6910071	32 315 077	6.89E-95	4.11E-169	9.82E-07	4.98E-77	2.99E-11	5.21E-67
rs531094	32 318 325	4.46E-53	4.35E-95	2.55E-05	5.01E-43	1.19E-06	4.06E-35
rs539703	32 320 685	5.11E-53	6.65E-95	2.72E-05	3.19E-43	1.15E-06	3.08E-35
rs546857	32 321 463	1.80E-54	1.60E-97	1.47E-05	1.14E-42	4.93E-07	8.41E-37
rs547077	32 321 541	1.27E-53	1.06E-95	6.04E-05	1.57E-43	1.38E-06	7.50E-36
rs570963	32 321 817	1.18E-09	1.19E-16	2.47E-03	7.35E-12	2.16E-02	3.01E-06
rs9268235	32 322 431	9.68E-07	1.60E-10	4.24E-02	1.81E-06	1.11E-04	2.04E-09
rs521828	32 323 866	4.11E-26	3.25E-43	2.20E-03	1.52E-26	3.01E-05	1.66E-24
rs523627	32 324 060	3.73E-53	2.64E-95	2.55E-05	5.01E-43	1.32E-06	5.37E-35
rs525607	32 324 307	1.06E-53	6.43E-96	6.04E-05	1.57E-43	1.50E-06	9.93E-36
rs552339	32 324 938	4.19E-53	3.27E-95	2.55E-05	5.01E-43	1.32E-06	5.37E-35
rs504203	32 327 066	4.08E-53	2.95E-95	2.51E-05	3.99E-43	1.37E-06	7.21E-35
rs505274	32 327 215	3.86E-53	2.59E-95	2.51E-05	3.99E-43	1.49E-06	7.35E-35
rs508805	32 327 573	4.50E-53	3.38E-95	2.51E-05	3.99E-43	1.51E-06	9.35E-35
rs524578	32 327 580	1.15E-53	7.18E-96	6.03E-05	1.25E-43	1.54E-06	1.34E-35
rs9348880	32 329 003	1.37E-53	8.51E-96	6.67E-05	2.59E-43	1.56E-06	1.02E-35
rs9348881	32 329 065	1.33E-54	2.67E-97	6.95E-05	2.39E-43	1.56E-06	1.02E-35
rs9368713	32 329 560	2.17E-53	7.31E-96	2.74E-05	4.21E-43	1.52E-06	5.64E-35
rs9405090	32 330 595	6.25E-53	4.85E-95	2.85E-05	8.24E-43	1.38E-06	5.52E-35
rs3129949	32 331 037	9.55E-18	7.23E-30	2.23E-03	1.33E-20	4.17E-05	2.54E-17
rs1003878	32 332 045	8.77E-18	6.61E-30	2.23E-03	1.33E-20	4.17E-05	2.54E-17
rs910052	32 332 096	5.30E-53	4.02E-95	2.85E-05	8.24E-43	1.38E-06	5.52E-35
rs9366793	32 333 512	1.37E-53	8.51E-96	6.67E-05	2.59E-43	1.64E-06	1.05E-35
rs9348882	32 333 545	5.14E-53	3.96E-95	2.85E-05	8.24E-43	1.38E-06	5.52E-35
rs9380290	32 333 675	1.33E-53	8.41E-96	6.67E-05	2.59E-43	1.56E-06	1.02E-35

rs9357140	32 333 737	6.40E-53	5.11E-95	2.91E-05	1.11E-42	1.53E-06	7.35E-35
rs1474729	32 334 268	6.35E-53	4.69E-95	2.47E-05	5.20E-43	1.38E-06	5.52E-35
rs1474728	32 334 290	6.05E-53	4.80E-95	2.69E-05	6.46E-43	1.22E-06	4.97E-35
rs1265757	32 334 605	9.40E-07	1.60E-10	4.24E-02	1.81E-06	1.11E-04	2.04E-09
rs10807100	32 335 291	5.30E-53	4.02E-95	2.85E-05	8.24E-43	1.29E-06	5.13E-35
rs6929776	32 335 734	1.37E-53	8.51E-96	6.75E-05	2.79E-43	1.56E-06	1.02E-35
rs1265754	32 335 915	9.98E-07	1.72E-10	4.62E-02	1.69E-06	1.21E-04	2.40E-09
rs28361060	32 336 071	9.27E-95	1.90E-169	8.61E-07	6.34E-77	2.66E-11	3.54E-67
rs6930681	32 336 254	5.30E-53	4.02E-95	2.85E-05	8.24E-43	1.38E-06	5.52E-35
rs926594	32 337 593	5.70E-53	4.92E-95	2.67E-05	6.67E-43	1.38E-06	5.52E-35
rs9368716	32 338 313	8.41E-33	4.90E-60	1.54E-04	1.27E-27	1.24E-04	6.55E-28
rs2022534	32 339 360	5.30E-53	4.02E-95	2.78E-05	8.36E-43	9.84E-07	7.13E-35
rs2022533	32 339 483	1.37E-53	8.51E-96	6.67E-05	2.59E-43	1.06E-06	1.32E-35
rs1033500	32 339 605	4.21E-53	2.52E-95	2.88E-05	8.63E-43	4.05E-07	3.92E-35
rs2143468	32 341 226	1.44E-50	9.44E-91	5.30E-05	2.42E-41	6.35E-07	1.28E-33
rs2143466	32 341 546	1.21E-50	1.38E-89	1.76E-05	2.20E-40	9.57E-07	6.93E-34
rs2143465	32 341 575	5.30E-53	4.02E-95	2.85E-05	8.24E-43	1.07E-06	7.19E-35
rs3117137	32 342 134	8.34E-18	9.95E-30	2.02E-03	1.31E-20	4.17E-05	2.54E-17
rs9268260	32 343 190	5.30E-53	4.02E-95	2.85E-05	8.24E-43	9.84E-07	7.13E-35
rs4959094	32 345 359	6.22E-53	5.35E-95	2.85E-05	8.24E-43	1.03E-06	9.12E-35
rs4959025	32 345 366	6.22E-53	5.35E-95	2.85E-05	8.24E-43	9.84E-07	7.13E-35
rs4959026	32 345 684	6.17E-53	5.68E-95	2.85E-05	8.24E-43	9.04E-07	5.12E-35
rs4959096	32 346 899	5.40E-53	4.50E-95	2.92E-05	1.04E-42	9.84E-07	7.13E-35
rs9268283	32 347 396	5.64E-53	4.88E-95	2.92E-05	1.04E-42	9.84E-07	7.13E-35
rs9268284	32 347 439	5.40E-53	4.50E-95	2.92E-05	1.04E-42	1.01E-06	9.20E-35
rs9268285	32 347 780	5.40E-53	4.50E-95	2.92E-05	1.04E-42	9.84E-07	7.13E-35
rs2076542	32 349 350	7.13E-53	6.73E-95	2.93E-05	2.74E-42	9.35E-07	4.92E-35
rs2076541	32 349 438	5.40E-53	4.50E-95	2.98E-05	1.33E-42	1.04E-06	7.18E-35

rs2076540	32 349 499	2.29E-54	1.76E-97	1.57E-05	1.87E-42	4.12E-07	1.50E-36
rs2076538	32 349 694	6.22E-53	5.35E-95	2.85E-05	8.24E-43	9.84E-07	7.13E-35
rs761187	32 350 259	2.97E-54	2.58E-97	1.62E-05	2.35E-42	3.97E-07	1.24E-36
rs3117119	32 350 833	3.14E-16	1.79E-28	5.96E-03	8.93E-12	2.81E-02	1.07E-07
rs3132963	32 352 376	2.90E-16	1.71E-28	3.54E-03	5.00E-12	2.74E-02	1.14E-07
rs2395149	32 357 785	9.40E-07	1.60E-10	4.24E-02	1.81E-06	1.11E-04	2.04E-09
rs9268326	32 362 376	5.30E-53	4.02E-95	2.74E-05	8.61E-43	9.84E-07	7.13E-35
rs9268343	32 364 047	6.12E-53	7.18E-95	3.35E-05	2.30E-42	9.84E-07	7.13E-35
rs9268345	32 364 064	5.30E-53	4.02E-95	2.73E-05	6.68E-43	9.84E-07	7.13E-35
rs9268362	32 365 564	2.30E-95	1.55E-169	8.53E-07	8.61E-78	3.84E-11	8.63E-67
rs3129927	32 366 050	9.40E-07	1.60E-10	4.24E-02	1.81E-06	1.54E-04	3.26E-09
rs9268368	32 366 178	5.30E-53	4.02E-95	2.73E-05	6.68E-43	9.84E-07	7.13E-35
rs2073046	32 368 297	5.30E-53	4.02E-95	2.73E-05	6.68E-43	9.84E-07	7.13E-35
rs3129938	32 368 718	3.18E-16	1.92E-28	6.03E-03	6.64E-12	2.91E-02	1.46E-07
rs9268384	32 368 809	5.30E-53	4.02E-95	2.78E-05	5.82E-43	7.76E-07	3.85E-35
rs3129943	32 370 918	2.23E-19	1.43E-32	9.71E-04	1.38E-20	1.52E-05	5.58E-20
rs2073044	32 371 209	1.46E-79	1.50E-141	2.14E-08	2.18E-69	2.45E-12	2.84E-62
rs2076536	32 371 571	2.77E-41	3.10E-72	3.86E-08	1.64E-40	1.50E-04	8.47E-25
rs9268401	32 373 541	2.94E-65	4.66E-119	8.87E-06	4.31E-53	6.82E-09	2.69E-56
rs9268403	32 373 696	4.00E-65	6.67E-119	8.87E-06	4.31E-53	7.36E-09	1.95E-56
rs9268404	32 373 942	2.73E-65	3.15E-119	8.04E-06	2.67E-53	5.97E-09	1.26E-56
rs9268428	32 377 196	3.28E-65	5.21E-119	8.87E-06	4.31E-53	7.36E-09	1.95E-56
rs2395153	32 377 818	9.62E-82	1.57E-137	2.43E-15	3.56E-64	2.27E-19	1.35E-65
rs9268456	32 382 169	3.57E-65	6.81E-119	1.02E-05	6.59E-53	7.08E-09	2.13E-56
rs9268459	32 382 999	4.18E-65	7.29E-119	9.51E-06	4.81E-53	6.31E-09	2.44E-56
rs4373382	32 383 091	4.44E-72	1.35E-121	7.67E-12	3.27E-54	1.83E-13	1.53E-55
rs4424066	32 386 651	2.05E-72	4.34E-122	1.19E-11	9.68E-54	7.40E-13	2.57E-54
rs3129948	32 386 867	3.60E-30	6.64E-53	7.18E-05	7.01E-28	7.31E-05	1.73E-15

rs9268472	32 387 828	1.10E-72	1.12E-122	7.74E-12	3.35E-54	1.86E-13	3.01E-55
rs9268474	32 389 388	9.38E-65	2.38E-118	8.71E-06	2.41E-53	7.36E-09	1.95E-56
rs3129950	32 390 424	2.49E-07	8.57E-12	2.65E-02	1.03E-07	1.50E-04	4.33E-11
rs9268475	32 390 454	9.38E-65	2.38E-118	8.71E-06	2.41E-53	7.36E-09	1.95E-56
rs3117098	32 390 736	6.21E-30	1.54E-52	6.14E-05	4.98E-28	7.11E-05	1.96E-15
rs17577980	32 392 044	1.39E-07	1.71E-15	1.71E-09	3.85E-13	4.44E-03	8.91E-06
rs3817973	32 393 334	2.61E-72	3.52E-122	7.23E-12	1.77E-54	2.36E-13	2.94E-55
rs2076530	32 396 039	1.81E-70	3.53E-119	2.54E-12	1.75E-55	2.47E-13	5.18E-54
rs9268480	32 396 067	5.06E-65	1.30E-118	8.71E-06	2.41E-53	7.36E-09	1.95E-56
rs2076529	32 396 178	1.28E-72	1.52E-122	7.23E-12	1.77E-54	2.36E-13	2.94E-55
rs28366191	32 396 413	4.88E-10	8.61E-18	3.92E-04	1.11E-10	5.19E-03	2.22E-05
rs9268481	32 396 579	5.06E-65	1.30E-118	8.71E-06	2.41E-53	6.89E-09	8.73E-57
rs3129954	32 397 803	3.54E-30	6.11E-53	6.01E-05	6.41E-28	7.11E-05	1.96E-15
rs3129955	32 398 063	9.55E-31	7.11E-54	6.70E-05	6.46E-28	1.06E-04	1.36E-14
rs2294880	32 399 945	2.77E-64	1.92E-117	4.76E-06	1.45E-53	5.68E-09	9.79E-57
rs9268482	32 400 000	5.06E-65	1.30E-118	8.71E-06	2.41E-53	7.36E-09	1.95E-56
rs2294878	32 400 018	1.71E-63	9.84E-109	6.98E-12	1.66E-48	8.36E-08	1.02E-43
rs3817966	32 400 070	3.52E-65	2.56E-118	5.83E-06	5.75E-52	3.08E-09	1.17E-57
rs3817963	32 400 310	3.52E-65	2.56E-118	4.99E-06	3.78E-52	3.08E-09	1.17E-57
rs3817962	32 400 537	5.55E-65	1.44E-118	1.21E-05	3.16E-53	7.36E-09	1.95E-56
rs2076525	32 402 839	5.28E-65	1.50E-118	8.73E-06	3.52E-53	6.64E-09	1.49E-56
rs2076523	32 403 058	8.97E-44	2.49E-81	6.58E-05	4.15E-34	1.92E-08	2.24E-32
rs2076522	32 403 402	7.35E-65	2.65E-118	9.27E-06	3.02E-53	8.36E-09	1.31E-56
rs3793127	32 404 138	8.77E-88	1.47E-180	4.26E-07	7.26E-78	1.34E-09	2.31E-67
rs10947260	32 405 408	3.74E-09	1.98E-16	1.74E-02	1.92E-11	6.82E-16	2.20E-15
rs10947261	32 405 455	2.70E-08	2.14E-15	8.81E-03	4.72E-11	4.46E-14	8.55E-14
rs10947262	32 405 535	2.76E-08	2.22E-15	8.69E-03	3.48E-11	4.46E-14	8.55E-14
rs3806156	32 405 921	9.33E-44	2.50E-81	5.94E-05	4.56E-34	1.63E-08	2.01E-32

rs3806157	32 406 024	1.70E-43	2.82E-81	7.36E-05	6.12E-35	3.37E-08	1.36E-31
rs3763307	32 406 845	5.28E-65	1.50E-118	8.71E-06	2.41E-53	8.09E-09	1.06E-56
rs3763308	32 406 863	1.74E-05	1.91E-10	7.60E-03	1.45E-08	1.12E-08	5.93E-09
rs9268494	32 407 575	6.71E-68	1.30E-122	6.67E-07	5.89E-58	3.81E-11	2.74E-65
rs9268497	32 407 647	4.51E-68	9.95E-123	6.32E-07	5.92E-58	4.27E-11	4.62E-65
rs9268499	32 407 918	1.16E-62	5.79E-114	8.65E-06	3.73E-51	2.26E-09	4.57E-58
rs6926737	32 407 968	2.52E-63	4.67E-108	1.14E-09	2.86E-58	5.24E-11	6.52E-56
rs3763311	32 408 399	2.07E-62	2.41E-114	1.53E-05	2.94E-52	5.08E-09	6.98E-57
rs9268507	32 409 762	1.28E-63	1.70E-108	2.05E-09	2.44E-58	6.22E-11	5.92E-56
rs5007265	32 411 089	8.93E-64	9.82E-109	2.00E-09	1.71E-58	5.69E-11	4.49E-56
rs3129961	32 411 163	1.15E-30	1.73E-53	8.26E-05	4.68E-28	7.46E-05	1.59E-15
rs5007263	32 411 205	8.93E-64	9.82E-109	2.00E-09	1.71E-58	5.69E-11	4.49E-56
rs5007259	32 411 324	8.93E-64	9.82E-109	2.05E-09	2.44E-58	5.69E-11	4.49E-56
rs9268515	32 411 518	1.82E-82	8.63E-199	2.24E-05	8.34E-81	1.48E-08	5.04E-71
rs9268516	32 411 712	1.28E-63	3.41E-116	1.31E-05	2.70E-53	1.48E-08	1.35E-56
rs17208888	32 411 729	9.46E-08	9.71E-16	7.28E-10	1.81E-13	4.16E-03	5.94E-06
rs6932542	32 412 485	1.98E-63	3.75E-108	1.91E-09	3.33E-58	5.43E-11	4.17E-56
rs9279640	32 412 623	9.53E-31	1.38E-53	8.26E-05	4.68E-28	7.46E-05	1.59E-15
rs4502931	32 413 005	1.13E-63	1.93E-108	2.13E-09	9.44E-58	1.78E-11	1.24E-55
rs9268528	32 415 331	2.11E-53	5.56E-98	5.15E-04	1.75E-37	3.93E-08	7.46E-42
rs9268541	32 416 750	1.83E-08	1.64E-14	6.46E-04	5.25E-09	1.40E-03	1.16E-05
rs9268542	32 416 944	9.91E-52	2.07E-95	6.14E-04	2.08E-36	8.49E-08	3.65E-40
rs9268543	32 417 024	3.66E-83	1.63E-208	6.07E-05	1.88E-89	9.08E-08	6.14E-73
rs9268556	32 419 187	9.91E-52	2.07E-95	6.14E-04	2.08E-36	8.49E-08	3.65E-40
rs2395163	32 420 032	1.48E-89	2.18E-186	2.23E-07	7.66E-86	7.96E-09	1.40E-67
rs9268557	32 421 528	7.59E-73	6.35E-123	1.76E-06	4.01E-52	1.36E-09	6.56E-55
rs3135363	32 421 871	8.75E-24	1.36E-43	5.75E-04	2.39E-32	8.45E-06	2.06E-34
rs2187818	32 427 791	3.45E-58	8.17E-104	8.05E-04	7.29E-38	1.50E-10	4.52E-43

rs9268585	32 429 626	3.18E-58	7.13E-104	7.82E-04	7.96E-38	1.54E-10	5.20E-43
rs9268589	32 430 425	2.82E-58	2.69E-104	8.85E-04	2.25E-37	5.87E-11	5.18E-43
rs9268606	32 432 293	2.33E-58	2.55E-104	8.91E-04	2.88E-37	5.87E-11	5.18E-43
rs984778	32 432 311	7.64E-48	1.02E-81	2.86E-02	6.92E-33	1.54E-03	6.53E-24
rs9501626	32 432 567	1.57E-16	3.87E-28	2.25E-02	4.34E-14	1.33E-08	2.98E-13
rs3135338	32 433 440	5.01E-48	5.01E-82	2.90E-02	5.85E-33	1.53E-03	6.32E-24
rs3135335	32 434 068	5.58E-48	6.01E-82	2.90E-02	7.96E-33	1.50E-03	6.87E-24
rs2027856	32 434 928	1.57E-16	3.87E-28	2.25E-02	4.34E-14	1.94E-08	3.79E-13
rs9268614	32 435 001	1.63E-91	3.02E-182	2.62E-07	1.57E-85	8.71E-09	1.59E-67
rs9268615	32 435 112	7.43E-59	7.82E-105	9.35E-04	4.22E-37	2.74E-10	1.45E-42
rs3129868	32 436 600	4.53E-16	7.93E-27	2.90E-03	2.10E-11	1.73E-03	1.43E-06
rs2395173	32 437 082	1.22E-47	1.85E-81	2.83E-02	4.73E-33	1.53E-03	6.32E-24
rs9268626	32 437 267	2.37E-06	7.85E-12	7.48E-07	5.12E-11	1.95E-02	9.82E-04
rs3135395	32 437 415	5.71E-48	5.82E-82	2.86E-02	6.92E-33	1.53E-03	6.32E-24
rs9268628	32 438 088	7.36E-08	8.41E-18	3.62E-09	5.12E-19	6.15E-03	8.87E-07
rs9268633	32 438 696	2.41E-21	3.74E-37	9.38E-04	3.24E-16	3.74E-04	4.66E-09
rs14004	32 439 932	4.83E-57	1.34E-101	3.46E-04	2.97E-37	6.84E-10	2.26E-42
rs9268644	32 440 267	1.45E-16	7.87E-31	3.78E-04	1.22E-10	1.42E-02	2.56E-18
rs9268645	32 440 750	4.95E-58	1.43E-103	1.10E-03	3.03E-37	8.47E-11	5.77E-43
rs3135392	32 441 465	5.38E-11	5.01E-20	2.25E-02	9.38E-08	2.64E-04	9.40E-20
rs3129882	32 441 753	1.59E-40	5.32E-74	1.60E-08	2.72E-42	8.30E-06	8.09E-26
rs9268657	32 441 879	5.85E-58	3.93E-103	1.16E-03	1.99E-37	1.58E-10	6.84E-43
rs6931646	32 442 004	1.25E-35	6.52E-65	2.33E-03	8.57E-24	1.72E-07	3.42E-35
rs6911419	32 442 010	2.05E-35	1.31E-64	2.36E-03	1.08E-23	1.76E-07	4.57E-35
rs6911777	32 442 219	7.19E-10	9.77E-17	3.30E-03	3.03E-10	4.93E-03	1.83E-05
rs3129886	32 442 799	3.44E-33	1.07E-56	3.55E-04	1.03E-22	2.84E-04	8.55E-19
rs9268658	32 442 939	1.36E-35	8.02E-65	2.33E-03	8.57E-24	1.60E-07	2.60E-35
rs3135391	32 443 210	3.36E-17	4.37E-28	4.17E-03	5.12E-12	5.61E-04	2.74E-07

rs8084	32 443 258	2.99E-65	2.15E-108	3.24E-06	2.41E-48	3.37E-11	3.88E-57
rs2239804	32 443 746	1.23E-35	7.29E-65	2.33E-03	8.57E-24	1.72E-07	3.42E-35
rs7192	32 443 869	1.57E-57	3.85E-95	3.67E-04	8.26E-44	3.33E-09	1.13E-52
rs3129888	32 443 949	5.87E-26	3.12E-43	3.61E-02	3.94E-17	5.50E-04	4.93E-16
rs2239803	32 444 056	2.49E-40	2.07E-73	2.02E-03	8.81E-30	8.75E-09	1.75E-41
rs2239802	32 444 069	2.64E-28	7.05E-48	4.94E-03	8.59E-20	6.59E-04	4.03E-16
rs7195	32 444 762	3.20E-57	1.25E-94	5.45E-04	1.11E-43	1.36E-09	1.40E-53
rs7197	32 444 803	1.76E-22	1.64E-37	3.68E-02	2.45E-16	3.66E-03	4.80E-13
rs3135388	32 445 274	3.35E-17	5.13E-28	8.71E-03	9.29E-12	5.43E-04	2.26E-07
rs2213586	32 445 317	1.28E-57	2.95E-95	4.86E-04	6.30E-44	3.84E-09	3.80E-53
rs2213585	32 445 373	1.43E-57	3.30E-95	4.90E-04	7.83E-44	3.84E-09	3.80E-53
rs2395182	32 445 540	2.50E-28	6.93E-48	5.83E-03	4.31E-20	7.69E-04	7.24E-16
rs2227139	32 445 682	1.20E-57	2.42E-95	5.12E-04	9.75E-44	3.84E-09	3.80E-53
rs3129889	32 445 768	3.35E-17	5.13E-28	8.71E-03	9.29E-12	5.43E-04	2.26E-07
rs3763326	32 445 780	1.09E-07	2.93E-12	3.21E-04	7.50E-07	5.97E-09	3.21E-09
rs3763327	32 446 053	1.75E-57	5.05E-95	3.05E-04	6.18E-43	3.06E-09	1.18E-52
rs7754768	32 452 402	4.44E-56	3.22E-93	2.06E-04	2.48E-42	5.85E-09	7.91E-54
rs6457590	32 454 021	9.18E-10	1.23E-17	3.33E-02	6.93E-10	3.52E-05	1.16E-09
rs9268831	32 459 971	6.56E-24	2.19E-45	3.19E-03	1.50E-16	1.14E-03	5.05E-23
rs9268832	32 460 012	1.19E-55	1.13E-92	5.83E-05	9.48E-42	2.11E-09	9.18E-54
rs9268835	32 460 338	3.95E-81	3.76E-143	1.55E-07	4.79E-73	2.23E-11	3.46E-64
rs6923504	32 460 409	3.47E-39	7.55E-70	4.01E-06	4.27E-46	4.52E-06	2.87E-21
rs6903608	32 460 508	3.63E-39	8.59E-70	3.98E-06	5.92E-46	4.52E-06	2.87E-21
rs9268838	32 460 938	4.00E-81	4.48E-143	1.46E-07	2.45E-73	2.47E-11	1.91E-64
rs9268853	32 461 866	8.17E-67	2.50E-120	1.02E-06	3.95E-64	1.50E-11	2.86E-57
rs9268882	32 463 846	4.40E-39	1.12E-69	3.17E-06	1.66E-46	5.02E-06	2.16E-21
rs9268923	32 465 058	4.31E-67	1.07E-120	9.84E-07	2.84E-64	1.69E-11	3.76E-57
rs2395185	32 465 390	6.08E-67	1.66E-120	9.84E-07	2.84E-64	1.69E-11	3.76E-57

rs9405108	32 470 871	4.62E-67	9.73E-121	9.84E-07	2.84E-64	1.69E-11	3.76E-57
rs9269081	32 473 323	1.85E-41	6.47E-73	3.73E-06	2.85E-48	7.85E-06	6.22E-21
rs1964995	32 481 634	2.99E-103	5.31E-166	1.42E-15	9.64E-92	4.85E-19	2.15E-79
rs2157339	32 543 895	9.40E-10	1.73E-16	4.60E-02	2.07E-07	9.38E-10	5.19E-11
rs477515	32 601 914	5.82E-63	5.81E-115	7.87E-07	2.08E-60	2.09E-10	4.09E-57
rs9270986	32 606 283	2.68E-15	1.01E-25	1.62E-03	3.94E-10	1.18E-03	2.99E-07
rs2858867	32 607 548	2.06E-34	2.15E-60	5.98E-05	9.08E-37	8.24E-05	1.48E-32
rs482044	32 608 287	6.89E-38	8.07E-73	7.28E-06	1.39E-37	1.09E-08	5.52E-36
rs660895	32 609 603	5.20E-95	5.19E-217	2.11E-07	1.15E-100	1.17E-10	4.69E-68
rs532098	32 610 275	1.08E-23	8.75E-47	2.29E-03	1.61E-25	1.38E-02	8.85E-15
rs13207945	32 611 931	1.09E-23	9.84E-47	2.20E-03	1.71E-25	1.38E-02	8.85E-15
rs4530903	32 614 112	1.28E-05	8.09E-09	1.11E-02	3.91E-05	4.10E-02	6.03E-04
rs521539	32 614 196	5.64E-95	5.73E-217	2.11E-07	1.15E-100	1.29E-10	1.57E-68
rs642093	32 614 298	4.00E-18	1.48E-34	3.67E-04	5.21E-28	1.15E-02	1.15E-18
rs3104413	32 614 873	7.82E-97	6.55E-242	4.56E-07	3.50E-110	7.80E-11	8.49E-86
rs6931277	32 615 580	1.23E-96	4.23E-242	2.90E-07	8.41E-110	7.06E-11	2.47E-86
rs9271366	32 619 077	3.90E-17	1.63E-28	8.04E-04	1.29E-11	1.72E-03	8.43E-07
rs9271488	32 621 223	6.58E-68	1.91E-122	1.09E-07	3.92E-65	5.53E-11	4.44E-57
rs3129763	32 623 148	1.14E-20	3.03E-38	6.22E-05	3.88E-30	6.38E-03	2.56E-20
rs9271588	32 623 176	2.54E-87	2.51E-144	9.91E-13	1.12E-79	3.67E-15	1.35E-72
rs9271640	32 624 423	3.70E-19	4.07E-32	5.75E-03	1.09E-13	5.92E-03	4.02E-13
rs9271775	32 626 551	2.29E-21	2.56E-35	8.20E-03	6.84E-15	3.53E-03	7.87E-13
rs3129768	32 627 306	3.68E-19	4.06E-32	5.83E-03	1.29E-13	5.92E-03	4.02E-13
rs3104389	32 627 320	7.66E-68	2.37E-122	1.04E-07	3.06E-65	3.56E-11	4.62E-57
rs9271858	32 627 446	6.31E-27	1.66E-52	1.50E-05	3.92E-34	6.64E-04	1.07E-26
rs9272105	32 632 222	1.50E-31	1.63E-60	5.41E-07	1.48E-38	2.87E-07	5.38E-36
rs9272143	32 633 026	2.86E-26	1.48E-51	1.57E-05	4.02E-34	7.18E-04	9.75E-27
rs9272219	32 634 492	7.11E-27	2.03E-49	7.24E-04	6.17E-36	6.27E-04	1.27E-27

rs9273012	32 643 864	7.11E-27	2.03E-49	6.22E-04	3.16E-36	6.38E-04	1.12E-27
rs9273363	32 658 495	6.55E-11	1.11E-22	1.15E-03	1.23E-13	1.31E-02	6.35E-13
rs9273448	32 659 970	4.73E-42	1.83E-70	1.40E-03	2.01E-33	5.06E-07	2.08E-31
rs3891175	32 666 690	1.22E-24	3.44E-42	2.48E-03	5.22E-21	2.37E-06	2.48E-21
rs3852215	32 667 724	2.60E-21	3.21E-36	5.83E-03	2.65E-18	1.91E-06	1.10E-17
rs9275141	32 683 340	7.96E-34	1.24E-62	3.57E-03	1.91E-26	5.61E-06	2.94E-31
rs3021061	32 684 062	6.44E-13	2.63E-26	9.23E-05	4.58E-18	8.41E-03	1.76E-22
rs4947342	32 685 293	4.13E-13	9.02E-27	4.74E-05	3.99E-19	8.41E-03	1.76E-22
rs4642516	32 689 766	7.96E-34	1.24E-62	4.60E-03	1.37E-26	4.37E-06	3.73E-31
rs7775228	32 690 302	2.48E-16	4.56E-29	1.92E-02	1.52E-11	2.31E-07	4.13E-13
rs9275224	32 692 101	2.53E-97	6.24E-158	3.53E-09	3.27E-70	6.43E-12	4.06E-65
rs2858324	32 692 598	1.41E-65	1.17E-108	3.78E-06	3.40E-53	5.08E-10	3.18E-55
rs5000634	32 695 787	1.17E-61	8.67E-110	7.45E-04	5.11E-44	9.10E-09	3.64E-40
rs6457617	32 696 074	9.04E-96	3.33E-156	9.14E-09	8.57E-70	1.41E-12	1.44E-64
rs6457620	32 696 222	1.10E-95	4.32E-156	5.55E-09	5.33E-70	1.44E-12	7.73E-65
rs2647015	32 696 316	2.28E-12	1.95E-22	7.01E-03	7.01E-11	8.59E-03	7.96E-06
rs7745040	32 696 555	1.17E-61	8.67E-110	7.38E-04	3.73E-44	1.71E-08	9.44E-40
rs2647012	32 696 681	3.16E-66	9.55E-110	5.22E-06	2.35E-52	8.04E-10	3.03E-55
rs9275332	32 699 166	2.73E-60	4.50E-107	9.18E-08	5.82E-56	1.47E-12	5.60E-47
rs9275334	32 699 330	6.78E-52	2.47E-118	2.82E-06	1.18E-53	1.47E-03	6.07E-51
rs9275338	32 699 566	2.25E-45	3.64E-92	5.38E-06	8.07E-47	1.47E-03	3.10E-34
rs17427599	32 699 587	1.74E-41	4.10E-74	1.17E-05	1.68E-33	7.08E-03	1.12E-15
rs9275371	32 700 519	2.92E-60	4.94E-107	1.14E-07	6.35E-56	4.28E-12	9.14E-46
rs1612904	32 701 241	5.55E-55	7.36E-93	8.73E-05	2.65E-46	1.58E-08	1.98E-51
rs9275390	32 701 379	8.02E-60	1.92E-106	8.18E-08	7.18E-56	1.39E-12	6.08E-47
rs9275393	32 701 662	2.62E-60	4.25E-107	1.16E-07	9.25E-56	1.36E-12	7.46E-47
rs2858308	32 702 223	8.22E-13	4.92E-23	1.08E-02	1.07E-10	3.93E-03	5.46E-06
rs9275407	32 702 260	2.73E-60	4.50E-107	9.18E-08	5.82E-56	1.36E-12	5.77E-47

rs2856717	32 702 531	2.50E-65	2.17E-108	1.02E-06	1.84E-52	7.01E-10	1.51E-54
rs9275425	32 703 097	2.86E-60	4.85E-107	1.64E-07	4.79E-56	1.38E-12	2.18E-46
rs2856705	32 703 179	9.77E-13	4.42E-23	7.00E-03	5.87E-11	4.86E-03	5.65E-06
rs9275428	32 703 201	2.73E-60	4.50E-107	9.18E-08	5.82E-56	1.39E-12	6.08E-47
rs9275439	32 703 744	3.35E-60	8.05E-107	9.18E-08	5.82E-56	1.39E-12	6.08E-47
rs9275495	32 705 797	8.53E-52	1.34E-117	3.75E-06	1.73E-53	1.47E-03	5.06E-51
rs9275530	32 707 746	1.80E-51	9.66E-118	3.81E-06	1.99E-53	1.30E-03	3.21E-50
rs9275532	32 707 857	9.16E-52	4.11E-118	2.47E-06	8.89E-54	1.47E-03	5.06E-51
rs9275580	32 711 685	4.33E-71	3.30E-124	1.86E-07	2.58E-60	7.46E-13	1.83E-60
rs3129727	32 711 913	1.47E-05	3.96E-09	7.96E-04	1.92E-06	1.10E-02	6.10E-04
rs9275582	32 712 293	7.87E-71	3.27E-124	1.17E-06	1.11E-57	6.18E-12	1.60E-60
rs7745656	32 713 193	2.63E-20	2.66E-40	5.83E-06	4.89E-24	1.20E-02	5.86E-12
rs2647087	32 713 272	2.28E-20	1.70E-40	5.55E-06	3.74E-24	1.20E-02	5.86E-12
rs2858332	32 713 384	3.30E-15	6.97E-26	1.05E-02	1.33E-12	2.76E-10	2.19E-28
rs3957146	32 713 753	1.33E-51	7.74E-118	2.48E-06	1.71E-53	1.31E-03	9.53E-51
rs2647089	32 713 791	1.87E-20	1.73E-40	5.60E-06	2.88E-24	1.17E-02	7.21E-12
rs9275596	32 713 854	2.96E-55	3.81E-93	6.41E-05	4.54E-45	1.64E-08	6.38E-50
rs6935723	32 713 892	2.94E-20	2.54E-40	5.55E-06	3.18E-24	1.11E-02	7.82E-12
rs3104402	32 713 899	1.26E-10	1.81E-17	2.56E-03	5.24E-05	3.27E-02	8.36E-06
rs3998158	32 714 215	2.76E-73	6.41E-127	7.78E-08	1.33E-63	5.73E-11	4.68E-60
rs3998159	32 714 242	3.49E-51	1.45E-116	5.36E-06	1.55E-52	1.25E-03	4.63E-51
rs9275599	32 714 652	8.41E-47	9.98E-106	1.53E-05	1.87E-49	3.31E-03	7.05E-48
rs3104407	32 714 675	8.04E-22	1.96E-41	6.35E-03	3.30E-19	9.82E-03	1.56E-20
rs3873444	32 714 947	1.12E-10	8.17E-22	1.04E-06	1.53E-16	2.81E-06	3.21E-08
rs9275698	32 720 196	3.06E-31	1.11E-54	3.28E-04	8.30E-21	3.44E-02	1.61E-14
rs9276189	32 731 548	1.03E-11	1.69E-19	4.89E-03	4.43E-07	2.24E-02	5.24E-05
rs4394270	32 742 187	1.19E-28	6.79E-50	9.95E-04	4.16E-16	1.59E-02	1.66E-14
rs2857208	32 775 326	4.91E-10	4.70E-18	2.96E-02	1.29E-08	1.52E-02	1.05E-04

rs7767167	32 797 405	2.29E-12	7.31E-21	9.93E-03	8.99E-09	5.55E-03	7.24E-06
rs2857107	32 817 738	2.80E-12	6.44E-22	8.38E-04	1.85E-12	1.11E-04	4.66E-07
rs1894411	32 825 196	2.36E-12	5.14E-22	8.77E-04	5.25E-12	1.63E-04	6.55E-07
rs3819721	32 837 021	3.68E-46	4.70E-84	1.91E-02	1.44E-27	5.31E-03	2.45E-22
rs9276831	32 864 256	1.89E-09	1.29E-18	1.26E-04	2.77E-11	1.10E-03	1.70E-06
rs9500927	32 993 584	9.93E-12	3.52E-23	6.04E-03	4.70E-13	2.36E-05	4.98E-09
rs9380335	33 057 397	1.72E-11	7.29E-22	2.73E-02	1.79E-12	3.22E-04	1.46E-08
rs4604307	33 057 894	2.14E-11	1.10E-21	2.88E-02	1.95E-12	2.04E-04	9.51E-09
rs2395309	33 058 469	1.65E-11	7.24E-22	2.81E-02	3.05E-12	2.84E-04	1.22E-08
rs7905	33 065 198	1.65E-09	8.57E-19	3.10E-03	4.47E-11	1.73E-03	5.25E-07
rs3077	33 065 245	1.84E-11	8.89E-22	2.73E-02	1.79E-12	2.84E-04	1.22E-08
rs9469332	33 067 099	1.63E-11	8.05E-22	1.86E-02	8.77E-13	2.84E-04	1.22E-08
rs9469341	33 068 100	1.77E-11	8.61E-22	1.99E-02	1.25E-12	2.77E-04	1.13E-08
rs1126769	33 068 657	1.99E-11	8.85E-22	2.88E-02	2.71E-12	2.84E-04	1.22E-08
rs10214910	33 069 898	2.43E-11	2.51E-21	1.40E-02	3.16E-13	1.39E-04	6.12E-09
rs2301224	33 070 592	1.65E-11	1.36E-21	4.24E-02	2.12E-12	8.97E-05	4.62E-09
rs2301220	33 070 989	1.31E-11	8.79E-22	4.01E-02	2.69E-12	7.62E-05	3.73E-09
rs6914849	33 072 938	1.51E-11	1.04E-21	4.01E-02	2.69E-12	6.78E-05	3.22E-09
rs9348904	33 073 058	1.51E-11	1.04E-21	4.12E-02	2.51E-12	7.55E-05	3.95E-09
rs1431399	33 073 257	1.51E-11	1.04E-21	4.01E-02	2.69E-12	7.62E-05	3.73E-09
rs1431400	33 073 399	1.61E-11	1.16E-21	4.01E-02	2.69E-12	7.29E-05	3.10E-09
rs1431401	33 073 409	1.51E-11	1.04E-21	4.01E-02	2.69E-12	7.29E-05	3.10E-09
rs987870	33 075 103	1.64E-07	1.43E-14	4.75E-03	3.96E-09	4.32E-04	4.89E-07
rs2071351	33 076 153	1.32E-11	9.02E-22	4.01E-02	2.69E-12	6.73E-05	3.61E-09
